# iRhom2 regulates ectodomain shedding and surface expression of the major histocompatibility complex (MHC) class I

**DOI:** 10.1007/s00018-024-05201-7

**Published:** 2024-04-04

**Authors:** Matteo Calligaris, Donatella P. Spanò, Simone Bonelli, Stephan A. Müller, Claudia Carcione, Danilo D’apolito, Giandomenico Amico, Monica Miele, Mariangela Di Bella, Giovanni Zito, Elisa Nuti, Armando Rossello, Carl P. Blobel, Stefan F. Lichtenthaler, Simone D. Scilabra

**Affiliations:** 1 Department of Research IRCCS ISMETT (Istituto Mediterraneo per i Trapianti e Terapie ad Alta Specializzazione), Proteomics Group of Ri.MED Foundation, via Ernesto Tricomi 5, 90127 Palermo, Italy; 2 Department of Pharmacy, University of Pisa, via Bonanno 6, 56126 Pisa, Italy; 3 STEBICEF (Dipartimento di Scienze e Tecnologie Biologiche Chimiche e Farmaceutiche), Università degli Studi di Palermo, Viale delle Scienze Ed. 16, 90128 Palermo, Italy; 4 German Center for Neurodegenerative Diseases (DZNE), Feodor-Lynen Strasse 17, 81377 Munich, Germany; 5 Neuroproteomics, School of Medicine, Klinikum rechts der Isar, Technische Universität München, 81675 Munich, Germany; 6 Department of Research IRCCS ISMETT (Istituto Mediterraneo per i Trapianti e Terapie ad Alta Specializzazione), Ri.MED Foundation, via Ernesto Tricomi 5, 90127 Palermo, Italy; 7 Department of Research, IRCCS ISMETT (Istituto Mediterraneo per i Trapianti e Terapie ad Alta Specializzazione), 90127 Palermo, Italy; 8 Arthritis and Tissue Degeneration Program, Hospital for Special Surgery, Program in Physiology, Biophysics and Systems Biology, Weill Cornell Medicine, New York, NY USA; 9 Institute for Advanced Study, Technical University Munich, Munich, Germany; 10 Munich Cluster for Systems Neurology (SyNergy), Munich, Germany

**Keywords:** iRhom2, ADAM17, Secretome, Macrophages, MHC class I molecules

## Abstract

Proteolytic release of transmembrane proteins from the cell surface, the so called ectodomain shedding, is a key process in inflammation. Inactive rhomboid 2 (iRhom2) plays a crucial role in this context, in that it guides maturation and function of the sheddase ADAM17 (a disintegrin and metalloproteinase 17) in immune cells, and, ultimately, its ability to release inflammatory mediators such as tumor necrosis factor α (TNFα). Yet, the macrophage sheddome of iRhom2/ADAM17, which is the collection of substrates that are released by the proteolytic complex, is only partly known. In this study, we applied high-resolution proteomics to murine and human iRhom2-deficient macrophages for a systematic identification of substrates, and therefore functions, of the iRhom2/ADAM17 proteolytic complex. We found that iRhom2 loss suppressed the release of a group of transmembrane proteins, including known (e.g. CSF1R) and putative novel ADAM17 substrates. In the latter group, shedding of major histocompatibility complex class I molecules (MHC-I) was consistently reduced in both murine and human macrophages when iRhom2 was ablated. Intriguingly, it emerged that in addition to its shedding, iRhom2 could also control surface expression of MHC-I by an undefined mechanism. We have demonstrated the biological significance of this process by using an in vitro model of CD8^+^ T-cell (CTL) activation. In this model, iRhom2 loss and consequent reduction of MHC-I expression on the cell surface of an Epstein-Barr virus (EBV)-transformed lymphoblastoid cell line dampened activation of autologous CTLs and their cell-mediated cytotoxicity. Taken together, this study uncovers a new role for iRhom2 in controlling cell surface levels of MHC-I by a dual mechanism that involves regulation of their surface expression and ectodomain shedding.

**Supplementary Information:**

The online version contains supplementary material available at 10.1007/s00018-024-05201-7.

## Introduction

ADAM17 is a member of the disintegrin metalloproteinase family of sheddases, a class of proteinases that releases transmembrane proteins from the cell surface [1–5]. Since its discovery as the tumor necrosis factor (TNF) α converting enzyme (TACE), ADAM17 has been reported to release over 50 different proteins, spanning from growth factors and cytokines, to signaling receptors and adhesion molecules [2, 5]. Consequently, ADAM17 orchestrates several biological processes, including cell-communication, adhesion and inflammation. Aberrant activity of ADAM17 is associated with a number of pathological conditions such as cancer and arthritis, indicating that its activity must be finely regulated to maintain homeostasis [2, 5]. In the last years, two inactive rhomboid pseudoproteases, known as iRhom1 and iRhom2, have emerged as essential regulators of ADAM17 in mammals [6]. iRhoms guide ADAM17 trafficking from the ER to the Golgi, where it gets activated by prodomain removal, and hence to the cell surface where ADAM17 cleaves its substrates [7–10]. Different mouse models have been used to investigate tissue expression and functions of iRhom1 and iRhom2 in vivo [7, 8]. While a study reported that both iRhoms are expressed in most mammalian tissues where they support maturation and function of ADAM17 [8], other studies reported that iRhom1 is generally expressed at higher levels than iRhom2, and that iRhom2 is upregulated by inflammatory stimuli [7, 11]. Interestingly, immune cells represent an exception to this common theme, with only iRhom2, but not iRhom1, being expressed [12]. In line, genetic ablation of iRhom2 in mouse does not cause developmental abnormalities, but leads to ADAM17 inactivation in immune cells. As a consequence, iRhom2-deficient mice have defects in releasing TNF and initiating innate immune responses against invading pathogens, but they are protected against diseases driven by excess inflammation, such as sepsis and rheumatoid arthritis [9, 10, 12]. Evidence has emerged that the function of iRhom1 and iRhom2 are not fully redundant. For instance, iRhom2 can control the substrate selectivity of ADAM17 [13, 14]. Thus, stimulated shedding of certain substrates occurs when the proteinase is in complex with iRhom2, but not with iRhom1. Furthermore, iRhom2 plays key functions in immunity that are not dependent on its ability to support ADAM17 maturation, including activation of immune responses to DNA and RNA viruses by regulating the stimulator of interferon genes (STING) or virus-induced signaling adaptor (VISA), respectively [15, 16]. In conclusion, functions of iRhom2 in immune cells may be more complex than aiding ADAM17 maturation, in that it could selectively address the proteinase towards specific substrates, or control key mechanisms in immunity other than TNF release and type I interferon (IFN-I) production.

In order to profile substrates shed by the iRhom2/ADAM17 complex in immune cells, and uncover potential additional functions of iRhom2 elicited independently from the proteolytic activity of ADAM17, herein, we applied murine and human iRhom2-deficient macrophages to an unbiased high-resolution proteomic workflow that has been specifically developed to analyse the secretome of macrophages [17]. By this means, we identified a subset of membrane proteins whose shedding is regulated by the iRhom2/ADAM17 complex. Furthermore, we found that iRhom2 plays a role in controlling expression of MHC-I molecules and their release from the cell surface. We observed that inactivation of iRhom2 in Epstein–Barr virus infected lymphoblastoid cells, but not ADAM17 inhibition, reduced their ability to activate autologous cytotoxic T lymphocytes (CTLs) [18], thus reinforcing the biological significance of iRhom2 in controlling surface levels of MHC-I molecules.

## Materials and methods

### Isolation of bone-marrow derived macrophages

Murine macrophages were differentiated in vitro as previously described [19]. In brief, 8–12 week old iRhom2 knockout (KO) or wild-type (WT) mice were sacrificed by carbon dioxide (CO_2_) inhalation, and femurs and tibia collected. After removing joints from the bones, femurs and tibia were flushed with 2 ml RPMI (Gibco, part of Thermo Fischer Scientific, Billings, Montana, United States). Cells were strained through a nylon mesh to remove debris and clumps, into a 15 ml centrifuge tube. Then, cells were resuspended in 15 ml complete RPMI, and centrifuged at 230 × *g* for 5 min. Cell pellet was resuspended in 1 ml of red blood cell lysis buffer (Sigma-Aldrich, St. Louis, Missouri, United States) for 3 min to remove erythrocytes, then diluted in 15 ml RPMI and centrifuged again at 230 × *g* for 5 min. Pellet was resuspended in RPMI, then 10^6^ cells were plated in a bacteriological Petri Dish with 10 ml complete RPMI supplemented with 100 ng/ml recombinant murine M-CSF (Peprotech, London, UK). After 1 week, cells were differentiated into macrophages (98% of cells were F4/80 positive–not shown) and then used for further analysis (secretome analysis and FACS).

### Secretome analysis of iRhom2 KO murine macrophages

#### Sample preparation for LC–MS/MS

iRhom2 KO or WT bone marrow-derived macrophages (BMDMs) were washed twice, before incubation with 10 ml serum-free RPMI supplemented with either 100 ng/ml LPS, 100 ng/ml LPS and 25 ng/ml PMA or 1 μg/ml LPS for 1 h. Alternatively, iRhom2 KO or WT BMDMs were incubated for 1 h with serum-free RPMI without any supplement, or with 100 ng/ml LPS and 25 ng/ml PMA for 6 h. Conditioned media were concentrated 10 times by using a 30 kDa Vivaspin column (Sartorius AG, Göttingen, Germany) and then applied to filter-assisted sample preparation (FASP [20]). In brief, protein concentration was measured by using a 660 nm protein BCA assay kit following the vendor’s instructions (Thermo Fischer Scientific, Waltham, United States). 25 μg proteins were reduced by the addition of 1 m Dithiothreitol (DTT, Thermo Fischer Scientific) in 100 mm Tris/HCl, 8 m urea pH 8.5 for 30 min at 37 °C. Proteins were then alkylated in 50 mm iodoacetamide (IAA, Thermo Fischer Scientific) for 5 min at room temperature and washed twice in 100 mm Tris/HCl, 8 m urea pH 8.0 at 14,000 × *g* for 30 min. proteins were digested with 0.2 μg LysC (Promega, Madison, United States) in 25 mm Tris/HCl, 2 m urea pH 8.0 overnight (enzyme to protein ratio 1:50) and with 0.1 μg trypsin (Promega) in 50 mm ammonium bicarbonate for 4 h (enzyme to protein ratio 1:100). Resulting peptides were desalted by stop-and-go extraction (STAGE) on reverse phase C18 (Supelco Analytical Products, part of Sigma-Aldrich, Bellefonte, US), and eluted in 40 μl of 60% acetonitrile in 0.1% formic acid [21]. Then, volume was reduced in a SpeedVac (Thermo Fisher Scientific) and the peptides resuspended in 20 μl of 0.1% formic acid. Peptide concentration was measured by a NanoDrop microvolume spectrophotometer (Thermo Fischer Scientific) and 1 μg peptides were applied to LC–MS/MS analysis.

#### LC–MS/MS analysis

To achieve high sensitivity, a nanoLC system (EASY-nLC 1000, Proxeon–part of Thermo Scientific) with an in-house packed C18 column (30 cm × 75 μm ID, ReproSil-Pur 120 C18-AQ, 1.9 μm, Dr. Maisch GmbH, Germany) was coupled online via a nanospray flex ion source equipped with a PRSO-V1 column oven (Sonation, Biberach an der Riß, Germany) to a Q-Exactive mass spectrometer (Thermo Fischer Scientific). Peptides were separated using a binary gradient of water and acetonitrile containing 0.1% formic acid at 50 °C column temperature, and their intensities quantified by using label-free quantification (LFQ) and data-dependent acquisition (DDA). Full MS scans were acquired at a resolution of 70,000 (m/z range: 300–1400; automatic gain control (AGC) target: 1 × 10^6^; max injection time 50 ms). The DDA was used on the 10 most intense peptide ions per full MS scan for peptide fragmentation. A dynamic exclusion of 120 s was used for peptide fragmentation.

#### Proteomic data analysis

The data was analysed by the software Maxquant (maxquant.org, Max-Planck Institute Munich) version 2.0.1.0. The MS data were searched against a reviewed canonical fasta database of Mus Musculus from UniProt (download: February 2023, 17,137 entries). Trypsin was defined as protease. Two missed cleavages were allowed for the database search. The option first search was used to recalibrate the peptide masses within a window of 20 ppm. For the main search peptide and peptide fragment mass tolerances were set to 4.5 and 20 ppm, respectively. Carbamidomethylation of cysteine was defined as a static modification. Acetylation of the protein N-terminus as well as oxidation of methionine set as variable modifications. Label free quantification (LFQ) of proteins required at least two ratio counts of unique peptides. Only unique peptides were used for quantification. The LFQ values were log2 transformed and a two-sided Student’s t-test was used to evaluate proteins statistically significantly regulated between iRhom2 KO and WT BMDMs. A *p*-value less than 0.05 was set as the significance threshold.

### QARIP (Quantitative analysis of regulated intramembrane proteolysis) analysis of detected peptides

Transmembrane proteins identified as potential ADAM17 substrates (i.e. whose extracellular levels increased in response to LPS and decreased in iRhom2-deficient BMDMs) were analysed by QARIP web server (http://webclu.bio.wzw.tum.de/qarip/), which matches identified peptides to the protein membrane topology [22].

### Analysis of H2-D1 and CSF1R levels on stimulated BMDMs

After differentiation, BMDMs were stimulated for 1 h with 100 ng/ml LPS and 25 ng/ml PMA, in the presence or absence of the metalloproteinase inhibitor TAPI-1 (10 μm–Sigma Aldrich). Then, cells were detached from culture plates with the cell-dissociation buffer (Thermo Fischer Scientific) and rinsed in PBS. 1 × 10^6^ cells were resuspended in 100 μl PBS and treated with 1 μg of mouse Fc Block (purified Rat Anti-Mouse CD16/CD32 antibody–BD Biosciences, East Rutherford, United States) for 20 min on ice. Then cells were stained with PE-conjugated anti-H2-D1 antibody (clone 28-14-8) and APC-conjugated anti-CSF1R (Thermo Fischer Scientific).

### Isolation PBMC-derived macrophages

Human blood samples, used in the present study for isolation of PBMCs, arise from healthy volunteers. This study was approved by our institute’s Ethics Committee, and informed consent obtained from all the voluntary participants. The anonymous blood donors received oral and written information about the possibility that their blood would be used for research purposes.

Human peripheral blood mononuclear cells (PBMCs) were isolated from venous blood by density gradient centrifugation on Lympholyte Cell Separation Media (Cedarlane Laboratories, Burlington, Canada). CD14 + monocytes were separated from PBMCs by immunomagnetic sorting using anti-CD14 magnetic microbeads (MACS CD14 Microbeads; Miltenyi Biotec, Bergisch Gladbach, Germany). Immunomagneting sorting efficiency was 98% according to flow cytometry analysis (data not shown). CD14 + monocytes were immediately subjected to macrophage differentiation as previously described [23]. Briefly, 2 × 10^6^ CD14 + monocytes were cultured in a 6–well plate with RPMI 1640 medium (Gibco) supplemented with 10% FBS (Gibco), 1% penicillin/streptomycin (Sigma-Aldrich), 10 mm HEPES (Gibco), and 1 mm l-glutamine (Lonza, Morrisville United States). Macrophage differentiation was performed with 50 μg/ml GM-CSF (Miltenyi Biotec) for 5 days, followed by 4 days of 20 μg/ml LPS + 200UI IFNγ (Miltenyi Biotec) treatment. Then, cells were used for further analysis including iRhom2 silencing, secretome analysis and flow cytometry.

### Secretome analysis of iRhom2 knockdown monocyte-derived macrophages

iRhom2 expression in differentiated macrophages was silenced with specific siRNAs. Cells were transfected with 50 nm Stealth RNAi duplex against iRhom2 [also known by gene name Rhbdf2 (HSS128594, HSS128595, HSS188104)] or Stealth RNAi siRNA Negative Controls, by using Lipofectamine RNAiMAX (Thermo Fisher Scientific) according to the manufacturer’s instructions. After 48 h, iRhom2 knockdown was evaluated by quantitative PCR; and iRhom2 silenced and control (CT) macrophages were washed, and stimulated with 100 ng/ml LPS and 25 ng/ml for 3 h in serum free medium. Conditioned media were collected and applied to FASP and STAGE-Tips, as described above.

#### LC–MS/MS and proteomic data analysis

1.5 μg peptides per each sample were applied to a Dionex Ultimate 3000 RSLCnano LC system which was coupled online via a Nanospray Flex Ion Source to a Q-Exactive mass spectrometer (Thermo Fischer Scientific). Peptides were separated on Acclaim PEPMap C18 column (50 cm × 75 μm ID, Thermo Scientific) by using a binary gradient of water and acetonitrile. Data-dependent acquisition (DDA) was used for label free quantification (LFQ), as described above. The MS data were searched against a reviewed canonical fasta database of Homo Sapiens from UniProt (downloaded on November 2020). The “match between runs” option was enabled with a match time window of 1.5 min. LFQ of proteins required at least one ratio count of unique peptides. Unique and razor peptides were used for quantification. Data normalization was enabled. The LFQ values were log2 transformed and a two-sided Student’s t-test was used to evaluate proteins statistically significantly regulated between iR2KD and CT macrophages. A *p*-value less than 0.05 was set as the significance threshold.

### Secretome/shedding analysis of LPS-stimulated THP-1 cells

Human monocytic THP-1 cell line (ATCC TIB-202, kindly provided by Dr Chiara Cipollina) was grown in complete RPMI 1640 medium supplemented with 10% FBS, 10 mm Hepes, 1 mm pyruvate and 1 mm l-glutamine. THP-1 cells were differentiated into macrophages with 5 ng/ml PMA for 48 h [24]. After differentiation, iRhom2 or ADAM17 was silenced by incubating these cells with 50 nm Stealth siRNA duplex for iRhom2 (HSS128594, HSS128595, HSS188104) or ADAM17 (HSS110434, HSS110435, HSS186181) (Thermo Fischer Scientific). Appropriate Stealth RNAi siRNA Negative Controls were used as a control for sequence independent effects following the treatment, according to manufacturer’s instructions. Then, cells were washed with serum-free RPMI 1640 and stimulated with 100 ng/ml LPS and 25 ng/ml PMA in serum free medium for 3 h, either in the presence or absence of 5 μm JG26 or 10 μm marimastat (Sigma Aldrich). JG26 is a hydroxamate-based inhibitor of ADAM17 that was proven specific over other metalloproteinases by using an in vitro assay based on the cleavage of ALCAM [25]. In a cell system, we found that JG26 is a selective ADAM17 inhibitor, which fully inhibits the protease at concentrations higher than 10 µm, but also partially inhibits ADAM10 (by about 50%) at same concentrations (data not shown). Conditioned media of JG26 or marimastat treated cells were collected and applied to the LC–MS/MS analysis as described above for PBMCs.

Alternatively, conditioned media were collected and proteins concentrated by precipitation with 5% (w/v) trichloroacetic acid (TCA) overnight at 4 °C. Proteins were resuspended in 50 μl of Leammli Sample Buffer (BioRad Hercules, United States) and loaded onto a polyacrylamide gel for SDS-PAGE electrophoresis and blotted to a polyvinylidene difluoride (PVDF) membrane for immunoblotting. Cells were collected in STET Lysis Buffer (10 mm Tris–HCl, 1 mm EDTA, 100 mm NaCl, 1% Triton X-100) containing 1 × proteinase inhibitor mixture and 10 mm 1–10 phenanthroline (Sigma Aldrich). After protein quantification, 20 μg protein were loaded onto polyacrylamide gels for SDS-PAGE electrophoresis and immunoblotting. After blocking with 5% non-fat milk (Sigma) in PBS with 0.1% Tween, the membranes were incubated with anti-HLA Class 1 ABC antibody (clone EMR8-5, from Abcam, Cambridge, UK), anti-ADAM17 (ab39162, Abcam), anti-calnexin (ADI-SPA-860-F, Enzo Life Science) or an anti-iRhom2 that we have recently developed in house [26]. After incubation with appropriate peroxidase-conjugated secondary antibodies (anti-mouse or anti-rabbit from Promega, or anti-rat from Thermo Fischer Scientific), protein bands were detected with ECL and acquired through a Chemidoc image analyser (BioRad). Quantification of band intensity was performed by using ImageLab (BioRad) and normalized to the mean of the original non-normalized control values. A two-sided Student’s t-test was used to evaluate a statistically significant regulation of HLA, with a *p*-value less than 0.05 that was set as the significance threshold.

### Secretome/flow cytometry analysis of iRhom1, iRhom2 or iRhom1/2 knockout MEFs

iRhom1 KO (iR1KO), iRhom2 KO (iR2KO), iRhom1/2 double KO (iR1/2 dKO) and WT MEFs were generated as previously described [8, 26]. MEFs were seeded in 6–well plates, in complete DMEM supplemented with 2 mm Glutamine and 10% FBS until confluence. Then, cells were washed and starved for 2 h, before incubating them with 2 ml serum free DMEM containing 25 ng/ml PMA for 3 h. Next, conditioned media were collected, subjected to FASP and STAGE-tips, and finally to LC–MS/MS and data analysis as described above for BMDMs. Alternatively, levels of H2-D1 on the cell surface were analysed by flow cytometry, by using a similar protocol to the previously described for BMDMs.

### RNA extraction and gene expression analysis

Total RNA from different cells (PBMCs, THP-1 cells and MEFs) was extracted with the miRNeasy Mini Kit and treated with DNAse (Qiagen, Hilden, Germany), according to the manufacturer’s instructions. About 100 ng/μl RNA was reverse-transcribed with the high-capacity RNA-to-cDNA kit (Applied Biosystems, part of Thermo Fisher Scientific). Real-time PCR was performed by using TaqMan Universal Master Mix II (Thermo Fisher Scientific, USA) and specific primers for human RHBDF2 (Hs00226277), ADAM17 (Hs0104195) and GAPDH (Hs02786624) (all from Thermo Fisher Scientific). Expression of mRNA was quantified by PCR using StepOnePlus Real-Time PCR System (Applied Biosystems, Thermo Fisher Scientific). GAPDH was used as a reference gene for the relative quantification, assessed by 2^−(ΔΔCT)^ calculation for each mRNA.

### Generation of iRhom2 CRISPR/Cas9 KO cell lines

CRISPR guide for iRhom2 (5′-GCATGCTGTCCTGCTCGCCA-3′, targeting exon 3) and not targeting control sequences (NTC, 5′-TCCGGAGCTTCTTTCAGTCAA-3′) were cloned into vector lentiCRISPRv2 (Addgene, cat. No. 52961) as previously described [27]. HEK293T packaging cells were used for CRISPR/Cas9 lentiviral particle production. Cells were seeded on 10 cm culture dish and incubated o.n. at normal growth condition (37 °C, 5% CO_2_, DMEM with 10% FBS, P/S, 1% l-Glutamine and 10 mm sodium pyruvate). 1 ml of Optimem was supplemented with 37 μl of Lipofectamine 3000 and mixed. In a second tube, 1 ml of Optimem was mixed with 13.3 μg of the packaging plasmid pxPAX2, 9 μg pcDNA3.1-VSVG and with 18 μg of lentiCRISPRv2-iRhom2 or lentiCRISPRv2-NTC. Lipofectamine and plasmid were mixed and let for 20 min at 37 °C. HEK293T were washed with PBS and medium was changed with Optimem 10% FBS prior to cell transfection. The transfection mix was added to the cells and incubated o.n. Then, the medium was changed with DMEM supplemented with 2%FBS, 1% P/S, 10 mm sodium butyrate and after 24 h the conditioned medium containing viral particles was collected, centrifuged to remove cell debris and used for lentiviral transduction.

500 μl of medium containing lentiviral particles were added to 500 μl THP-1 cell suspension, in the presence of 5 μg/ml polybrene. Cells were transferred onto a 6–well plate and spinfected by centrifuging cells at 1800 rpm for 2 h at 33 °C. After an o.n. incubation with the viral suspension, cells were collected, washed twice and incubated with growth media supplemented with 1 μg/ml of puromycin for selection of transduced cells. After selection, the generation of iRhom2KO cell lines was evaluated by Western blotting. A similar procedure was used to generate iRhom2 KO fibroblast-like HTB94 cells.

### Analysis of MHC-I trafficking in iRhom2 KO cells

iRhom2KO HTB94 cells and its WT counterparts were grown in 6–well plates and then treated with 10 µg/ml brefeldin A (Sigma-Aldrich, US) or monensin (BD GolgiStop™ Protein Transport Inhibitor containing monensin, BD Biosciences, US) for 3 h. Then, cells were harvested with TryPLE reagent (Gibco, part of Thermo Fisher Scientific) and processed for flow cytometry analysis. Briefly, HTB94 were stained with FITC anti-HLA Class I [W6/32], isotype IgG2a (Abcam, Cambridge, UK) or PE anti-HLA Class I [W6/32], isotype IgG2a (BioLegend, San Diego, CA) at room temperature for 15 min according to the manufacturer’s instruction, washed with PBS and then resuspended in 300 µl PBS. Cells were analysed with FACS Celesta SORP flow cytometer and FACS Diva software version 9.0 (BD Biosciences, CA, US).

### Deglycosylation assay

20 µg of iRhom2 KO and WT THP-1 cell lysate, or iRhom2 KO and WT HTB94 lysate, were treated with endoglycosidase H (Endo H, New England Biolabs, P0702) or Peptide-N-Glycosidase F (PNGase F, New England Biolabs, P0704) according to the manufacturer’s protocol. Afterwards, the samples were separated SDS–PAGE electrophoresis and analysed by Western blotting.

### Generation of EBV-transformed LCLs and their autologous EBV-specific T-cell clones

Human EBV-transformed lymphoblastoid cell lines (LCL) were obtained by infecting PBMCs with EBV, as previously described [28]. Briefly, EBV-transformed B95-8 cell line (85011419, Sigma–Aldrich Co., USA) was used for production of EBV containing supernatants. PBMCs from two healthy volunteers were isolated by Ficoll–Hypaque density gradient centrifugation and incubated with EBV-containing supernatant from the B95-8 to produce lymphoblastoid cell lines (LCLs). LCLs were cultured in RPMI supplemented with 10% HyClone FBS (HyClone Laboratories, Logan, United States) and 800 ng/ml of cyclosporin-A (Sandoz Pharmaceuticals, Basel, Switzerland) to inhibit T cell mediated killing of infected B-cells. Immortalized LCLs were then cultured in complete RPMI 1640 medium. iRhom2 KO and control LCLs were generated by using CRISPR-Cas9, using as similar protocol to that described for the generation of iRhom2 KO THP-1 cells. To generate EBV-specific T-cell clones, PBMCs were incubated with autologous B-LCLs (i.e. derived from PBMCs of the same volunteer), irradiated at 60 Gy at an effector-to stimulator (E/S) ratio of 40:1. After 10 days, viable cells were restimulated with irradiated LCLs (at 4:1 E/S ratio) and 5 days later, 20 U/ml recombinant human interleukin 2 (Chiron, Emeryville, United States) was added. The T lymphocytes were weekly restimulated with LCLs (at 4:1 E/S ratio) in the presence of IL-2 (20 U/ml). Starting from day + 28, T cells were assessed for flow cytometry and then cryopreserved in 10% dimethyl sulfoxide (DMSO) 40% PBS and 50% human serum albumin.

### T cell cytotoxicity assay

The ability of iRhom2 KO or WT LCLs to induce cytotoxic activity in their autologous T cell clones was evaluated by a chromium release assay. Autologous iRhom2 KO or WT target LCL cells, or allogenic LCLs taken as a negative control, were labelled with 100 μCi of ^51^Cr (200–500 mCi/mg Na_2_^51^CrO_4_—Perkin Elmer, Waltham, United States) at 37 °C for 18 h. ^51^Cr-labelled target cells (10^4^ cells/well) were incubated for 4 h with autologous T cells at different T-cell/target ratios (from 40:1 to 5:1). Upon T cell-induced death, radioactivity was released from target cells into the supernatant. 25 μl of supernatant from each well were measured in a β scintillation microplate counter (PerkinElmer). For each sample, three independent parameters were measured: the spontaneous release of ^51^Cr in the condition media in the absence of T cell activity; the maximum release of ^51^Cr resulting from physical destruction of the cell membrane; and the experimental release obtained by incubating target cells with autologous T cells. The per cent specific lysis was calculated by the formula:

% specific lysis = (test release−spontaneous release)/(maximum release−spontaneous release) × 100.

### Investigation of iRhom2/HLA interaction by molecular modelling and co-immunoprecipitation

Alphafold was used to predict an interaction between iRhom2 and HLA, as it was previously used to model the iRhom2/ADAM17 interaction [29]. For the co-immunoprecipitation of iRhom2 and HLA, 2.2 × 10^6^ cells were seeded in a 10 cm dish. Then, 10 µg of pcDNA3.1 plasmid containing HA tagged iRhom2, HA tagged inactive signal peptide peptidase-like 2 protease b (Sppl2b-D/A) or empty plasmid were transfected with 30 µl of Lipofectamine 3000 (Invitrogen). After 48 h, cells were lysed in 1 ml of lysis buffer (50 mm Hepes pH 7.4, 150 mm NaCl, 5 mm EGTA, 1% Glycerol, 1% Triton X100, 1.5 mm MgCl_2_, 10 mm 1,10-Phenanthroline) supplemented with complete protease inhibitor (Roche, Basel, CH). Cell lysates were cleared by centrifugation at 16,000 *g* for 20 min at 4 °C. 500 µl of cleared lysates were applied to agarose beads coupled with anti-HA antibodies (A2095, Sigma–Aldrich, US) and incubated o/n at 4 °C. Then, beads were washed 6 times in lysis buffer and incubated with 20 µl of Laemmli buffer at 65 °C for 20 min. Eluted proteins were loaded onto an acrylamide gel, separated by SDS-PAGE electrophoresis and then analysed by Western blotting using following antibodies: anti-ADAM17 (ab39162, Abcam, Cambridge, UK), anti-HA [HA7] (Sigma-Aldrich, St. Louis, MO, US), anti-HLA,ABC [EMR8-5] (ab70328, Abcam, Cambridge, UK) and anti-GAPDH (Code 5174, Cell Signaling, Danvers, Massachussets, US).

## Results

### Secretome analysis identifies iRhom2 as a major regulator of MHC-I shedding

**Fig. 1:**
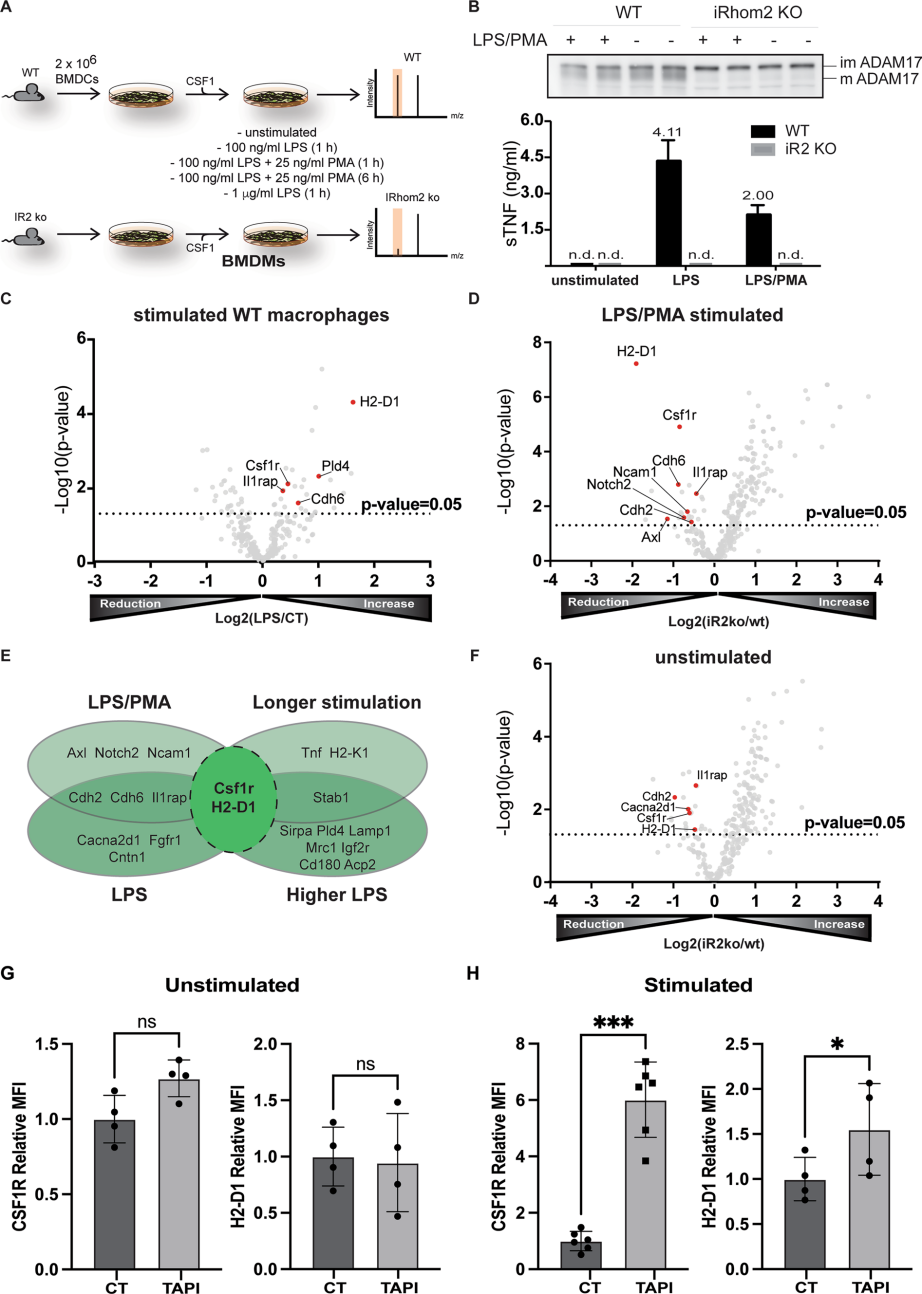
H2-D1 is shed in stimulated BMDMs. **A** Workflow for the secretome analysis of WT and iRhom2KO (iR2 KO) BMDMs subjected to various combinations of LPS and PMA. **B** iR2 KO or WT BMDMs were incubated for 1 h in the presence or absence of LPS or LPS/PMA and the release of TNF measured by ELISA (n = 3). A Western blot displays ADAM17 in the lysate of iRhom2 KO and WT BMDMs, in the presence or absence of LPS/PMA stimulation. While WT BMDMs clearly show two bands corresponding to the immature pro-enzyme (proADAM17) and mature active form of the proteinase, iRhom2 KO macrophages only show the band corresponding to proADAM17, as loss of iRhom2 prevents its maturation through the secretory pathway [9, 10]. **C** Volcano plot showing the –Log10 of *p*-values versus the log2 of protein ratio between LPS/PMA stimulated and control BMDMs (n = 5). The horizontal dotted line indicates the –log10 (*p*-value) of 1.3, which corresponds to a *p*-value of 0.05. Proteins above this line are considered significantly regulated. The ectodomain of a number of transmembrane proteins significantly increased upon stimulation. These proteins, including putative ADAM17 substrates, are displayed as red filled dots. Other proteins were displayed as gray dots. **D** Volcano plot showing the -Log10 of *p*-values versus the log2 of protein ratio between LPS/PMA treated iRhom2KO and WT BMDMs (n = 5). The ectodomain of various transmembrane proteins were significantly reduced in the secretome of iRhom2 KO compared to WT BMDMs. These proteins, putative ADAM17 substrates whose shedding depends on iRhom2, are displayed as red filled dots. Other proteins are displayed by filled gray dots. **E** Venn diagram showing the proteins annotated as transmembrane proteins that were reduced in the secretome of iRhom2 KO BMDMs upon different stimulations: 100 ng/ml LPS for 1 h (LPS); 100 ng/ml LPS and 25 ng/ml PMA for 1 h (LPS/PMA), 1 µg/ml LPS for 1 h (higher LPS); 100 ng/ml LPS and 25 ng/ml PMA for 6 h (longer stimulation). **F** Volcano plot showing the -Log10 of *p*-values versus the log2 of protein ratio between unstimulated iRhom2KO and WT BMDMs (n = 5). Transmembrane proteins of which the ectodomain was significantly reduced in iRhom2 KO BMDMs are displayed as red filled dots. Other proteins are displayed by filled gray dots. **G, H** Flow cytometry displaying that levels of CSF1R and H2-D1 are not affected by the metalloproteinase inhibitor TAPI in unstimulated BMDMs (**G**), but increase upon TAPI treatment in stimulated BMDMs (**H**) (shown as mean values ± standard deviation; *p < 0.05, ***p < 0.005, ns = non significant; Student’s t-test). Flow cytometry histograms of one representative experiment are shown in Supplemental Fig. 1G and H

iRhom2 has emerged as a major regulator of inflammatory responses due to its ability to trigger ADAM17-mediated release of TNF in immune cells [9, 10]. In order to investigate the role of iRhom2 in macrophages in a comprehensive manner, we set up a high-resolution proteomic workflow to analyse the secretome of murine bone marrow-derived macrophages (BMDMs) isolated from wild-type (WT) or iRhom2 KO (iR2KO) littermates (Fig. 1A). Monocytes were differentiated into macrophages prior stimulation for 1 h with LPS (100 ng/ml), either alone or in combination with the ADAM17 activator PMA [30] (25 ng/ml), with high-dose LPS (1 μg/ml) or with LPS and PMA for 6 h. Under these conditions ADAM17 gets activated and releases TNF and its other substrates [2, 31], and induces most of sheddome perturbations. In addition, macrophages were left unstimulated to analyse their constitutive shedding, which does not necessarily occur through ADAM17 and can involve other sheddases that are active in the absence of external stimuli (e.g. ADAM10 [32]). As expected, iR2KO macrophages were defective in expressing mature ADAM17 and releasing TNF in response to LPS or LPS/PMA (Fig. 1B). Secretome analysis detected 490 proteins in the conditioned media of LPS/PMA stimulated BMDMs, 110 more than in the conditioned media of unstimulated macrophages (Supplementary Tables S1). Among proteins found in the conditioned media of LPS/PMA stimulated BMDMs, 111 were classed as secreted and 163 as membrane proteins (based on Uniprot annotation), 20 secreted and 23 membrane proteins more than in unstimulated cells. In addition, the analysis identified 64 type 1, 12 type 2, 6 GPI-anchored and 60 membrane proteins with a different topology in the secretome of stimulated BMDMs (Supplementary Figure S1A). Levels of a number of transmembrane proteins increased upon stimulation, including (referred to by gene name) H2-D1, Il1rap, Cdh6, Pld4 and the iRhom2/ADAM17 substrate Csf1r [33] and Axl [34] (Table 1, Fig. 1C, Supplemental Table S1). Levels of TNF in the secretome of unstimulated macrophages were below the threshold of detection by mass spectrometry, while LPS stimulation for 1 h, either alone or in combination with PMA, promoted its release (Supplemental Figure S1B, Supplemental Table S1).

**Table 1:** List of the significantly (*p *value < 0.05) increased potentially shed proteins detected in the secretome of LPS/PMA stimulated BMDMs versus untreated controls

Protein names	Protein ID	Gene names	Peptides	Ratio	*p *value
H-2 class I histocompatibility antigen, D-B alpha chain	P01899	H2-D1	12	3.09	5.21E-05
Macrophage colony-stimulating factor 1 receptor	P09581	Csf1r	13	1.38	8.01E-03
Phospholipase D4	Q8BG07	Pld4	13	2.02	4.99E-03
Tyrosine-protein kinase receptor UFO	Q00993	Axl	5	3.06	1.18E-02
Interleukin-1 receptor accessory protein	Q61730	Il1rap	8	1.30	1.22E-02
Cadherin-6	P97326	Cdh6	6	1.57	2.58E-02

*Protein ID*: Uniprot accession number of the protein.

*Peptides*: number of identified unique peptides of the protein group.

*Ratio*: mean ratio of label-free quantification intensities between LPS/PMA stimulated BMDMs and unstimulated controls.

*p value*: for four biological replicates.

Upon iRhom2 ablation, levels of 163 proteins changed (with a *p*-value of their change below 0.05) in the secretome of LPS/PMA stimulated BMDMs (Fig. 1D and E), with levels of 8 transmembrane proteins being reduced: H2-D1, Csf1r, Il1rap, Cdh2, Cdh6, Axl, Ncam1, Notch2 (Table 2, Fig. 1D and E, Supplemental Table S1). TNF was only found in the secretome of stimulated WT macrophages, with no protein released by cells lacking iRhom2 and therefore functional ADAM17 (Supplemental Figure S1B, Supplemental Table S1). The ‘quantitative analysis of regulated intramembrane proteolysis’ (QARIP) webserver allows mapping identified peptides to the protein transmembrane topology [22]. QARIP analysis of iR2KO BMDM secretome revealed that the identified peptides of these reduced transmembrane proteins match only with their ectodomain, suggesting that these proteins underwent ADAM17-dependent shedding (Supplementary Figure S1C, Supplemental Table S1). Notably, our analysis did not detect IL-6R, L-selectin and other transmembrane proteins that are known to be shed by ADAM17 in immune cells. Although we cannot exclude that such ADAM17 substrates were not cleaved under these experimental conditions, we envisage that this mainly depended to the technical limitations of proteomics, including difficulties in the detection and quantification of low abundant proteins in proteomes with a large dynamic range such as conditioned media [35]. Previous reports displaying that iRhom2 controls shedding of IL-6R further supported the latter hypothesis [36].

**Table 2:** List of the significantly (*p* value < 0.05) reduced potentially shed proteins detected in the secretome of LPS/PMA stimulated iRhom2 KO BMDMs versus WT controls

Protein names	Protein ID	Gene names	Peptides	Ratio	*p *value
H-2 class I histocompatibility antigen. D-B alpha chain	P01899	H2-D1	12	0.27	5.97E-08
Macrophage colony-stimulating factor 1 receptor	P09581	Csf1r	13	0.56	1.23E-05
Cadherin-6	P97326	Cdh6	6	0.55	1.59E-03
Interleukin-1 receptor accessory protein	Q61730	Il1rap	8	0.74	3.43E-03
Neural cell adhesion molecule 1	P13595	Ncam1	10	0.64	1.58E-02
Neurogenic locus notch homolog protein 2	O35516	Notch2	4	0.60	2.62E-02
Tyrosine-protein kinase receptor UFO	Q00993	Axl	5	0.46	2.93E-02
Cadherin-2	P15116	Cdh2	7	0.68	3.79E-02

*Protein ID*: Uniprot accession number of the protein.

*Peptides*: number of identified unique peptides of the protein group.

*Ratio*: mean ratio of label-free quantification intensities between LPS/PMA stimulated BMDMs and unstimulated controls.

*p value*: for six biological replicates.

Stimulation of iR2KO and WT BMDMs with LPS led to similar results as LPS/PMA (Supplementary Figure S1D). In addition to TNF that was only released by WT cells, ablation of iRhom2 reduced shedding of 8 transmembrane proteins: H2-D1, Csf1r, Il1rap, Cdh2, Cdh6, Cacna2d1, Cntn1 and Fgfr1 (Fig. 1E, Supplemental Figure S1B and D, Supplemental Table S1). Likewise, a longer 6 h LPS/PMA stimulation showed reduction of TNF, H2-D1, H2-K1, Csf1r and Stab1 in the secretome of iR2KO macrophages compared to WT controls (Fig. 1E, Supplemental Figure S1E, Supplemental Table S1). Stimulation with high-dose LPS (1 μg/ml) had similar effects as low-dose LPS on the secretome of iR2KO BMDMs, with H2-D1 and Csf1r, being the most reduced proteins compared to WT BMDMs (Fig. 1E, Supplemental Figure S1F, Supplemental Table S1). Moreover, levels of an additional group of proteins were reduced, including Sirpa, CD180 and Lamp1, suggesting that their shedding could be affected by lack of iRhom2. Finally, secretome analysis of unstimulated iR2KO BMDMs found that levels of a number of proteins including Csf1r, H2-D1, Il1rap, Cacna2d1 and Cdh2 were reduced compared to WT BMDMs (Table 3, Fig. 1F, Supplemental Tables S1).

**Table 3:** List of the significantly (*p* value < 0.05) reduced proteins detected in the secretome of iRhom2 KO BMDMs versus WT controls in steady-state cnditions

Protein names	Protein ID	Gene names	Peptides	Ratio	*p* value
Interleukin-1 receptor accessory protein	Q61730	Il1rap	8	0.28	2.39E-06
Cadherin-2	P15116	Cdh2	7	0.58	3.92E-05
Voltage-dependent calcium channel subunit alpha-2/delta-1	O08532	Cacna2d1	14	0.72	7.01E-03
Macrophage colony-stimulating factor 1 receptor	P09581	Csf1r	13	0.71	1.65E-02
H-2 class I histocompatibility antigen. D-B alpha chain	P01899	H2-D1	12	0.7	2.30E-02

*Protein ID*: Uniprot accession number of the protein.

*Peptides*: number of identified unique peptides of the protein group.

*Ratio*: mean ratio of label-free quantification intensities between LPS/PMA stimulated BMDMs and unstimulated controls.

*p value*: for six biological replicates.

Together with the ectodomain of the known ADAM17 substrate CSF1R [33], extracellular levels of ectodomain of the class I histocompatibility antigen, H2-D1, increased upon LPS/PMA stimulation and were reduced by loss of iRhom2 in all datasets, suggesting it as a novel iRhom2/ADAM17 substrate in immune cells. Thus, we aimed to validate ADAM17-dependent ectodomain shedding of H2-D1 by using a synthetic inhibitor of ADAM metalloproteinases [37]. However, due to lack of suitable antibodies to detect shed H2-D1 in the conditioned media, we used flow cytometry to measure levels of H2-D1 on the cell surface of macrophages. In general, shedding inhibition reduces ectodomain levels in the extracellular space and, concomitantly, leads to an accumulation of transmembrane proteins at the cell membrane. In line, inhibition of ADAM-mediated shedding by TAPI-1 led to the increase of both Csf1r and H2-D1 in LPS/PMA stimulated macrophages, while it did not affect their cell membrane levels under steady-state conditions, in which ADAM17 is mostly inactive (Fig. 1G and H, Supplemental Fig. 1G and H). This further indicated that Csf1r and H2-D1 were shed by activated iRhom2/ADAM17 in BMDMs.

### iRhom2 regulates MHC-I shedding in human macrophages

**Fig. 2:**
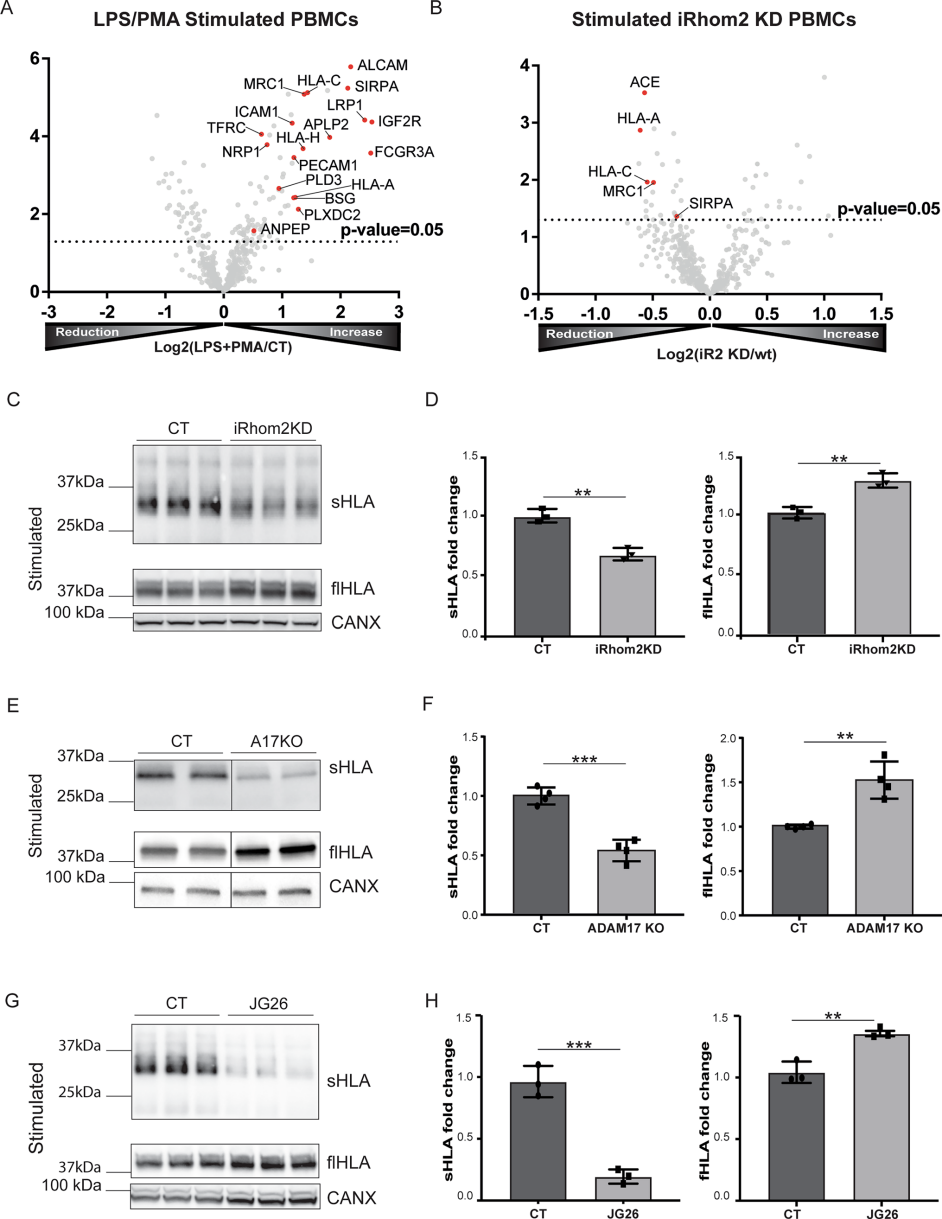
iRhom2/ADAM17 regulate MHC-I shedding in human macrophages. **A** Volcano plot showing the -Log10 of *p*-values versus the log2 of protein ratio between LPS/PMA stimulated and control human PBMC-derived macrophages. The horizontal dotted line indicates the –log10(*p*-value) of 1.3, which corresponds to a *p*-value of 0.05. Proteins above this line are considered significantly regulated. The ectodomain of a number transmembrane proteins were significantly increased upon stimulation. These proteins, which includes putative ADAM17 substrates, are displayed as red filled dots. Other proteins were displayed as gray dots. **B** Volcano plot showing the -Log10 of *p*-values versus the log2 of protein ratio between LPS/PMA treated iRhom2KD and control (CT) macrophages. Transmembrane proteins of which the ectodomain was significantly reduced in iRhom2KD cells are displayed as red filled dots. Other proteins are displayed by filled gray dots. **C, D** Immunoblots (**C**) and their respective quantification (**D**) showing levels of soluble HLA (sHLA) in the conditioned media and full-length HLA (flHLA) of LPS/PMA stimulated iRhom2 knock-down (iRhom2KD) or control (CT) macrophages (mRNA levels of iRhom2 after silencing are shown in the Supplemental Fig. 2A). While levels of shed HLA in the conditioned media of iRhom2KD macrophages decreased, levels of HLA in the cell lysate of iRhom2KD macrophages increased compared to control macrophages, indicating that iRhom2 silencing reduced shedding of HLA in LPS stimulated macrophages. **E, F** Similarly, immunoblots (**E**) and their respective quantification (**F**) show that levels of shed HLA (sHLA) in the conditioned media of LPS/PMA stimulated ADAM17 KO (A17KO) were reduced compared to control (CT) macrophages, while levels of HLA in the cell lysate (full length HLA–flHLA) were increased. Loss of ADAM17 in these cells is shown in Supplemental Fig. 2I. **G, H** Immunoblots (G) and their respective quantification (H) showing that levels of shed HLA (sHLA) in the conditioned media of LPS/PMA stimulated macrophages decreased when cells were treated with the ADAM17 selective inhibitor JG26 [25]. On the other hand, levels of HLA in the lysate (flHLA) increased upon treatment with JG26. Calnexin was used as a protein loading control. Densitometric quantifications shown as mean values ± standard deviation (*p < 0.05, **p < 0.01 ***p < 0.005; Student’s t-test)

Our proteomic analysis indicated that H2-D1 shedding was regulated by iRhom2. H2-D1 is the murine haplotype of MHC class I molecules (MHC-I) highly expressed in the C57BL/6 mouse, from which BMDMs were isolated. We investigated whether this mechanism was also conserved in human, where the human leukocyte antigen (HLA) system is expressed as MHC-I molecules. Thus, we stimulated wild-type human peripheral blood mononuclear cells (PBMCs)-derived macrophages with LPS/PMA and analysed the secretome by high-resolution mass spectrometry. In line with murine macrophages, stimulation increased shedding of a number of known ADAM17 substrates, including ALCAM, ICAM1, APLP2, PECAM1 and FCGR3A [2] (Fig. 2A, Table 4, Supplemental Table S2). Comparable with their murine counterparts, levels of shed HLA-A and HLA-C clearly increased in the secretome of human macrophages upon LPS/PMA stimulation (HLA-B was not detected). A number of ectodomains from transmembrane proteins were more abundant in the secretome of stimulated macrophages, including SIRPA, MRC1 and PLD3, suggesting that these could be novel putative substrates of ADAM17. Furthermore, knockdown of iRhom2 by siRNAs reduced the release of HLA-A and HLA-C in LPS/PMA stimulated PBMC-derived macrophages (Fig. 2B, Supplemental Fig. 2A, Supplemental Table S2), similar to ACE, MRC1 and SIRPA, transmembrane proteins whose ADAM17-mediated shedding could be dependent on iRhom2. In line, stimulation of macrophage-like THP-1 cells increased extracellular levels of HLA molecules, together with a number of ADAM17 substrates (e.g. TNF, ALCAM, AREG, etc.), as it was shown by unbiased proteomics (Supplemental Fig. 2B, Supplemental Table S2). Then, we confirmed that LPS/PMA stimulation increased shedding of HLA molecules by using immunoblotting as an orthogonal method (Supplemental Fig. 2C and 2D). Knockdown of iRhom2 in THP-1 cells reduced extracellular levels of HLA molecules (Fig. 2C and 2D, Supplemental Figure S2E). In stimulated THP-1 cells treatment with specific siRNAs only led to a partial knockdown of ADAM17, of about 60%, which was enough to reduce HLA release (Supplemental Figure S2F–H). Nevertheless, in order to confirm that such a reduction was due to lack of ADAM17-dependent shedding, we fully ablated ADAM17 in THP-1 cells by CRISPR-Cas9 (Supplemental Figure S2I). As expected, loss of ADAM17, as well as its chemical inhibition by a selective ADAM17 inhibitor (JG26 – [25]), diminished the release of HLA molecules in a similar manner as loss of iRhom2, (Fig. 2E–H). Mass spectrometry-based measurement further confirmed that released HLA-A and -C were reduced upon ADAM17 inhibition (Supplemental Figure S2L).

**Table 4:** List of the significantly (*p* value < 0.05) increased potentially shed proteins detected in the secretome of stimulated PBMC-derived macrophages versus untreated controls

Protein names	Protein ID	Gene names	Peptides	Ratio	*p *value
Tyrosine-protein phosphatase non-receptor type substrate 1	P78324	SIRPA	15	4.42	4.48E-06
CD166 antigen	Q13740	ALCAM	10	4.25	9.26E-06
HLA class I histocompatibility antigen, C alpha chain	Q95604	HLA-C	11	2.73	3.94E-05
Macrophage mannose receptor 1	P22897	MRC1	36	2.55	5.11E-05
Prolow-density lipoprotein receptor-related protein 1	Q07954	LRP1	20	5.58	8.06E-05
Intercellular adhesion molecule 1	P05362	ICAM1	9	2.16	1.76E-04
Cation-independent mannose-6-phosphate receptor	P11717	IGF2R	23	6.04	2.08E-04
Transferrin receptor protein 1	P02786	TFRC	14	1.60	2.44E-04
Neuropilin-1	O14786	NRP1	6	1.78	2.89E-04
Low affinity immunoglobulin gamma Fc region receptor III-A	P08637	FCGR3A	5	4.50	3.11E-04
Amyloid-like protein 2	Q06481	APLP2	8	3.92	4.00E-04
Putative HLA class I histocompatibility antigen, alpha chain H	P01893	HLA-H	5	2.51	7.94E-04
HLA class I histocompatibility antigen, A alpha chain	P01891	HLA-A	10	2.91	1.20E-03
Platelet endothelial cell adhesion molecule	P16284	PECAM1	13	2.52	1.22E-03
Phospholipase D3	Q8IV08	PLD3	5	2.10	1.39E-03
Basigin	P35613	BSG	4	2.14	8.80E-03
Aminopeptidase N	P15144	ANPEP	12	1.56	1.31E-02
Plexin domain-containing protein 2	Q6UX71	PLXDC2	5	2.32	1.81E-02

*Protein ID*: Uniprot accession number of the protein.

*Peptides*: number of identified unique peptides of the protein group.

*Ratio*: mean ratio of label-free quantification intensities between LPS/PMA stimulated BMDMs and unstimulated controls.

*p value*: for six biological replicates.

**Fig. 3:**
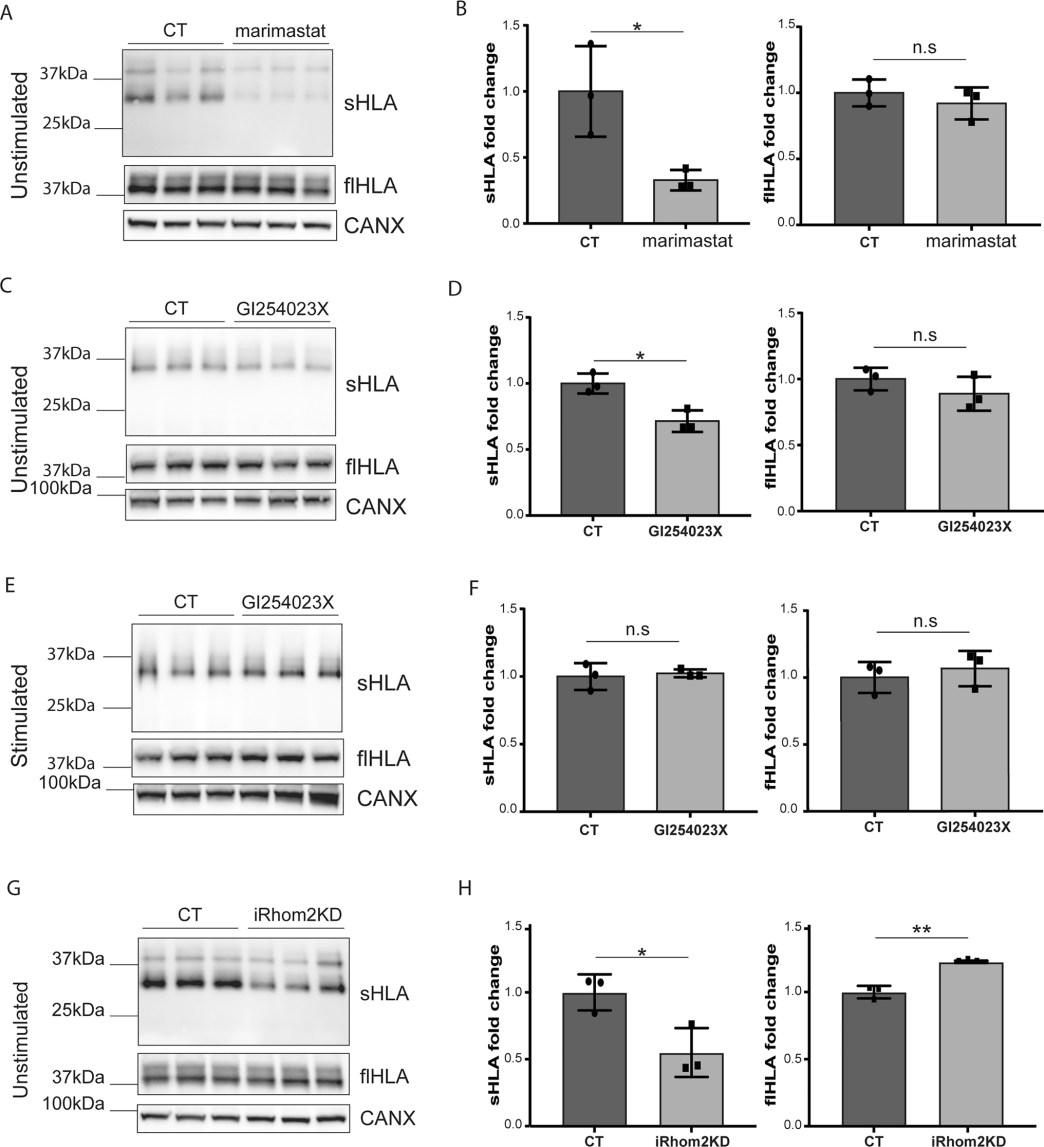
Constitutive HLA shedding is partly mediated by ADAM10. **A–D** Immunoblots and their respective quantification showing that marimastat (**A**, **B**) or the ADAM10 inhibitor GI254023X (**C**, **D**) decreased levels of shed HLA (sHLA) in the conditioned media of human PBMC-derived macrophages treated with the inhibitors for 24 h. Levels of HLA in the lysate (full length – flHLA) of marimastat or GI254023X treated macrophages did not change compared to untreated controls. **E**, **F** Conversely, immunoblots and their respective quantification show that levels of shed HLA (sHLA) in the conditioned media, as well as levels of HLA in the lysate (flHLA) of macrophages stimulated with LPS/PMA for 3 h were not altered in the presence or absence of GI254023X. **G, H** Immunoblots (**G**) and their respective quantification (**H**) showing that levels of shed HLA (sHLA) in the conditioned media of unstimulated iRhom2 knockdown (iRhom2KD) macrophages was reduced compared their control macrophages, and levels of HLA in the lysate (flHLA) increased. Calnexin (CANX) was used as a protein loading control. Densitometric quantifications shown as mean values ± standard deviation (*p < 0.05, **p < 0.01, n.s. non significant; Student’s t-test)

Secretome analysis of BMDMs found that MHC-I were also constitutively shed, and that loss of iRhom2 slightly decreased levels of shed H2-D1 in their secretome even in the absence of an ADAM17 activating stimulus (Fig. 1F). Thus, we analysed constitutive shedding of HLA molecules in THP-1 cells by incubating them with a synthetic inhibitor of metalloproteinases for 24 h and analysing levels of shed HLA in the conditioned media by immunoblotting. Metalloproteinase inhibition decreased levels of shed HLA in the conditioned media of THP-1 cells (Fig. 3A and B). Similarly, the ADAM10 inhibitor GI254023X partially reduced constitutive shedding of HLA in unstimulated THP-1 cells (Fig. 3C and D), indicating that the constitutive shedding of HLA in these cells involved ADAM10 and, potentially, other metalloproteinases. Conversely, the effect of GI254023X was negligible on LPS/PMA stimulated shedding of HLA in the same cells (Fig. 3E and F), suggesting that, upon stimulus, ADAM17 is the main sheddase of these proteins. Interestingly, iRhom2 knockdown reduced constitutive shedding of HLA molecules (Fig. 3G and H).

### iRhom2, but not iRhom1, regulates cell surface expression and shedding of MHC-I molecules

**Fig. 4:**
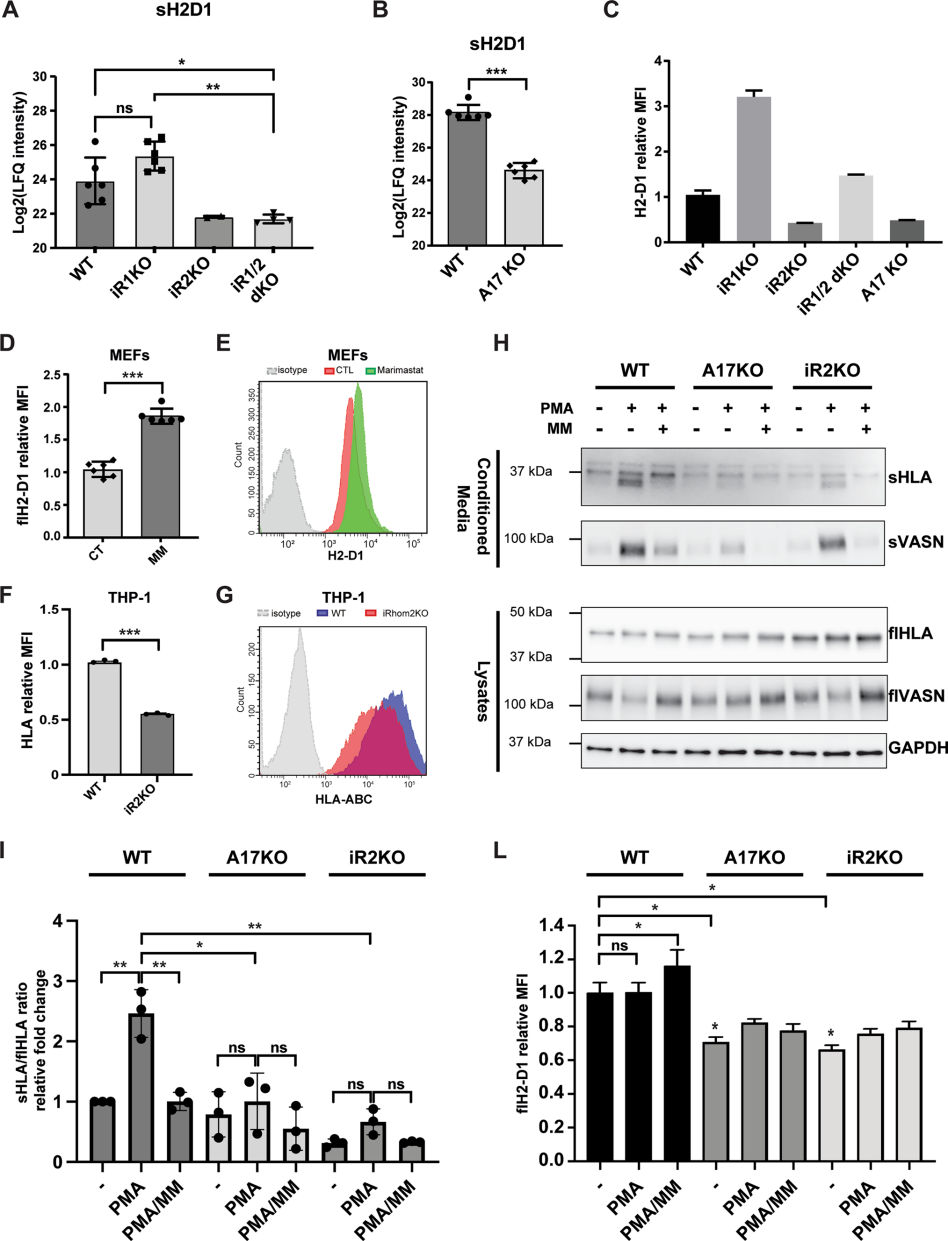
iRhom2 regulates membrane levels of MHC-I. **A, B** A mass spectrometry-based analysis followed by label-free quantification shows that levels of shed H2-D1 (sH2-D1) were not altered in the conditioned media of PMA-stimulated iR1KO MEFs compared to WT MEFs, while they were reduced in the conditioned media of iR2KO or iR1/2 dKO MEFs **A** (*p < 0.05, **p < 0.01, n.s. non significant; 2-way Anova). Of note: in 4 out of 6 iR2KO samples, levels of shed H2-D1 were below the detection threshold, and therefore a proper statistical analysis of the reduction of sH2-D1 in iR2KO samples could not be performed. **B** A similar analysis shows that levels of shed H2-D1 in the conditioned media of PMA-stimulated ADAM17 KO MEFs were reduced compared to WT controls (***p < 0.005, Student t-test). **C** Cell membrane levels of H2-D1 were measured by flow cytometry (n = 3) **D, E** Flow cytometry analysis shows that levels of H2-D1 on the cell surface of PMA-stimulated WT MEFs increased in the presence of 10 µm marimastat (MM) (***p < 0.005, Student t-test; flow cytometry histograms of one representative experiment are shown in (**E**). **F, G** Flow cytometry analysis shows that levels of HLA in iR2KO THP-1 cells were reduced compared to WT (***p < 0.005, Student t-test; flow cytometry histograms of one representative experiment are shown in (**G**). **H** Immunoblots show that levels of shed HLA (sHLA) increased in the conditioned media of PMA stimulated WT HTB94 cells compared to untreated controls. Addition of marimastat (MM) to PMA-stimulated cells reduced extracellular sHLA levels to those of unstimulated controls. sHLA in the conditioned media of stimulated A17KO or iR2KO cells was reduced compared to stimulated WT controls. Levels of full-length HLA (flHLA) in the lysate of A17KO cells and iR2KO slightly increased compared to WT cells. The ADAM17 substrate vasorin (VASN), is used as a control protein that is known to be shed in an ADAM17-dependent manner in PMA stimulated cells, and GAPDH is used as a protein loading control). **I** Bands of sHLA in the conditioned media were quantified and normalized to the corresponding flHLA bands in the lysates. Results from three independent experiments were displayed as the mean of fold change relative to untreated WT and analyzed by 2-way Anova (*p < 0.05; **p < 0.01, n.s. non-significant). **L** Flow cytometry shows that cell membrane levels of HLA increased when PMA stimulated WT HTB94 cells were treated with marimastat (MM), and that HLA levels decreased in ADAM17 KO and iRhom2 KO HTB94 compared to WT cells (*p < 0.05, n.s. non-significant; 2-way Anova)

MHC-I molecules are ubiquitously expressed, not only in immune cells, and therefore they are expressed in tissues where both iRhom1 and iRhom2 are present. It was reported that, other than supporting ADAM17 maturation, iRhoms can control its substrate selectivity [13, 14]. Thus, we investigated whether stimulated ADAM17-mediated shedding of MHC-I was only supported by iRhom2, or whether there was some redundancy with iRhom1. We stimulated wild type (WT), iRhom1 KO (iR1KO), iRhom2 KO (iR2KO), iRhom1 and iRhom2 double KO (iR1/2 dKO) mouse embryo fibroblasts MEFs and analysed H2-D1 levels in their conditioned media by mass spectrometry. While levels of shed H2-D1 in the conditioned media of PMA-stimulated iR1KO cells were comparable to WT, its levels were reduced in the conditioned media of iR2KO and iR1/2 dKO cells (Fig. 4A–of note: H2-D1 was detected only in 2 out of 6 biological replicates of iR2KO MEFs, while it was below the threshold of detectivity in the other 4 replicates). Similarly, levels of shed H2-D1 in the conditioned media of PMA-stimulated ADAM17 KO (A17KO) MEFs were reduced compared to WT MEFs (Fig. 4B). Furthermore, sH2-D1, which was clearly detected when WT cells were stimulated (LFQ intensity = 1.15 × 10^7^), it was below the threshold of detectivity in the same cells in the absence of PMA. PMA increased shedding of H2-D1 in iR1KO MEFs, but not in iR1/2 dKO or A17KO MEFs (Supplemental Fig. 3A), and it was not detected in either unstimulated and PMA-stimulated iR2KO MEFs. Then, to complement the shedding analysis, we assessed cell surface levels of H2-D1 in these cells, and, given that MEFs are an heterogenous population of cells isolated from the embryo trunk and the mesodermal tissues, we measured their H2-D1 expression by qPCR. We found that cell surface levels of H2-D1 in iR2KO and A17KO cells were lower than WT, despite its higher expression (Fig. 4C, Supplemental Fig. 3B). H2-D1 levels on the surface of iR1/2 dKO cells were comparable with WT, although they showed a higher number of transcripts. Both transcripts and surface levels of H2-D1 were higher in iR1KO cells than in WT. Altogether, these results indicated that iRhom2 loss could reduce H2-D1 at surface in post-transcriptional manner. In line with results on BMDMs (Fig. 1G and H), metalloproteinase inhibition led to opposite results than iRhom2 loss on MEF cell surface expression of H2-D1. When treated with marimastat, PMA-stimulated WT MEFs showed higher H2-D1 cell surface levels than controls (Fig. 4D and E). Loss of iRhom2 also had similar effects on the human counterpart of MHC-I, leading to decreased cell surface levels of HLA in human macrophage-like THP-1 cells (Fig. 4F and G). Altogether, these results suggested that, in addition to support its ADAM17-mediated shedding, iRhom2 could regulate cell surface levels of MHC-I, in mouse and human, by a different mechanism. Given the heterogeneity of MEF lines, to further confirm this hypothesis, we ablated ADAM17 and iRhom2 in a human fibroblast-like cell line (HTB94), in which both iRhom1 and iRhom2 are expressed (Supplementary Fig. 3D) and analysed HLA shedding and levels at the cell surface. PMA stimulation increased levels of HLA in the conditioned media of WT HTB94 cells, while metalloproteinase inhibition by marimastat reduced them (Fig. 4H and I), in line with levels of vasorin, a known ADAM17 substrate [38]. Loss of ADAM17 or iRhom2 clearly diminished extracellular levels of HLA in PMA-stimulated HTB94 cells compared to the WT controls. Then, we evaluated levels full length HLA in cell lysates by Western blot and on the cell membrane by flow cytometry. PMA stimulation, which increased HLA levels in the conditioned media, had negligible effects on its cell lysate or surface levels (Fig. 4H–L). Conversely, metalloproteinase inhibition did not change its levels in the lysate, but it slightly increased its cell surface levels, in line with metalloproteinase inhibition in murine fibroblasts (Fig. 4L). Akin to MEFs and THP-1 cells, loss of iRhom2 reduced surface levels of HLA in HTB94 cells, in spite of a mild overall increase in their cellular levels (Fig. 4H–L). ADAM17 ablation led to indistinguishable results from iR2KO HTB94 cells on HLA shedding and membrane expression. Indeed, ADAM17 is known to stabilize iRhom2, preventing it from degradation [26]. Similarly, ADAM17 ablation in HTB94 cells led to iRhom2 degradation (Supplemental Fig. 3D).

**Fig. 5:**
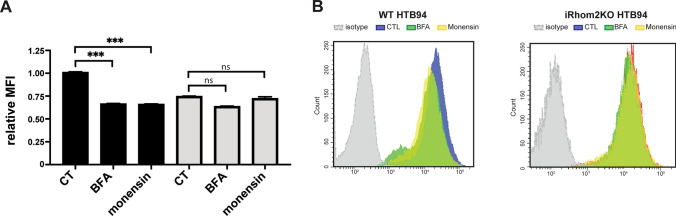
iRhom2 loss reduces cell surface levels of HLA. **A, B** Cell membrane levels of HLA in wild-type or iRhom2 KO HTB94 cells, treated with or without brefeldin A (BFA) or monensin and measured by flow cytometry (n = 3, 2-way Anova, ***p < 0.005). Flow cytometry histograms of one representative experiment are shown in **B**

In addition to ADAM17, iRhom2 was reported to support the trafficking of STING and VISA [15, 16]. We reasoned that iRhom2 could control surface levels of MHC-I by regulating its trafficking in a similar manner. Thus, we treated iR2KO and WT HTB94 cells with brefeldin A, which inhibits protein transport from the ER to the Golgi apparatus, or monensin, which prevents protein secretion from the medial to trans cisternae of the Golgi complex. Both drugs clearly reduced HLA levels at the surface of WT HTB94 cells, while they had minimal effect on iRhom2-deficient cells (Fig. 5A and B). Nevertheless, when we treated lysates of HTB94 cells with endoglycosidase H (EndoH), which cleaves off immature N-glycans that are added to proteins in the ER but not mature glycans after they are elaborated in the Golgi apparatus, HLA was entirely endo-H resistant and no evident differences emerged between iR2KO and WT endoH-treated cells (Supplemental Fig. 3E). In contrast, peptide-N-glycosidase F (PNGaseF), which removes almost all N-linked oligosaccharides from glycoproteins, led to complete deglycosylation of HLA in these cells. Similarly, when we treated lysates of THP-1 cells the majority of HLA was still endoH resistant (Supplemental Fig. 3F). These results show that HLA exit from the ER is quite rapid, and that iR2KO plays a minor role, if any, in HLA trafficking through the secretory pathway.

ΑlphaFold failed to predict any direct interaction between iRhom2 and HLA (Supplemental Fig. 4A) and overexpressed iRhom2 did not co-immunoprecipitate with HLA (Supplemental Fig. 4B). Altogether these data indicate that in addition to controlling its shedding, iRhom2 could also control cell membrane expression of MHC-I, in mouse and human, by a yet to be established mechanism that iRhom1 is not able to compensate.

### iRhom2 ablation KO cells evade CD8 + T cell immune response by affecting surface expression of MHC class I

**Fig. 6:**
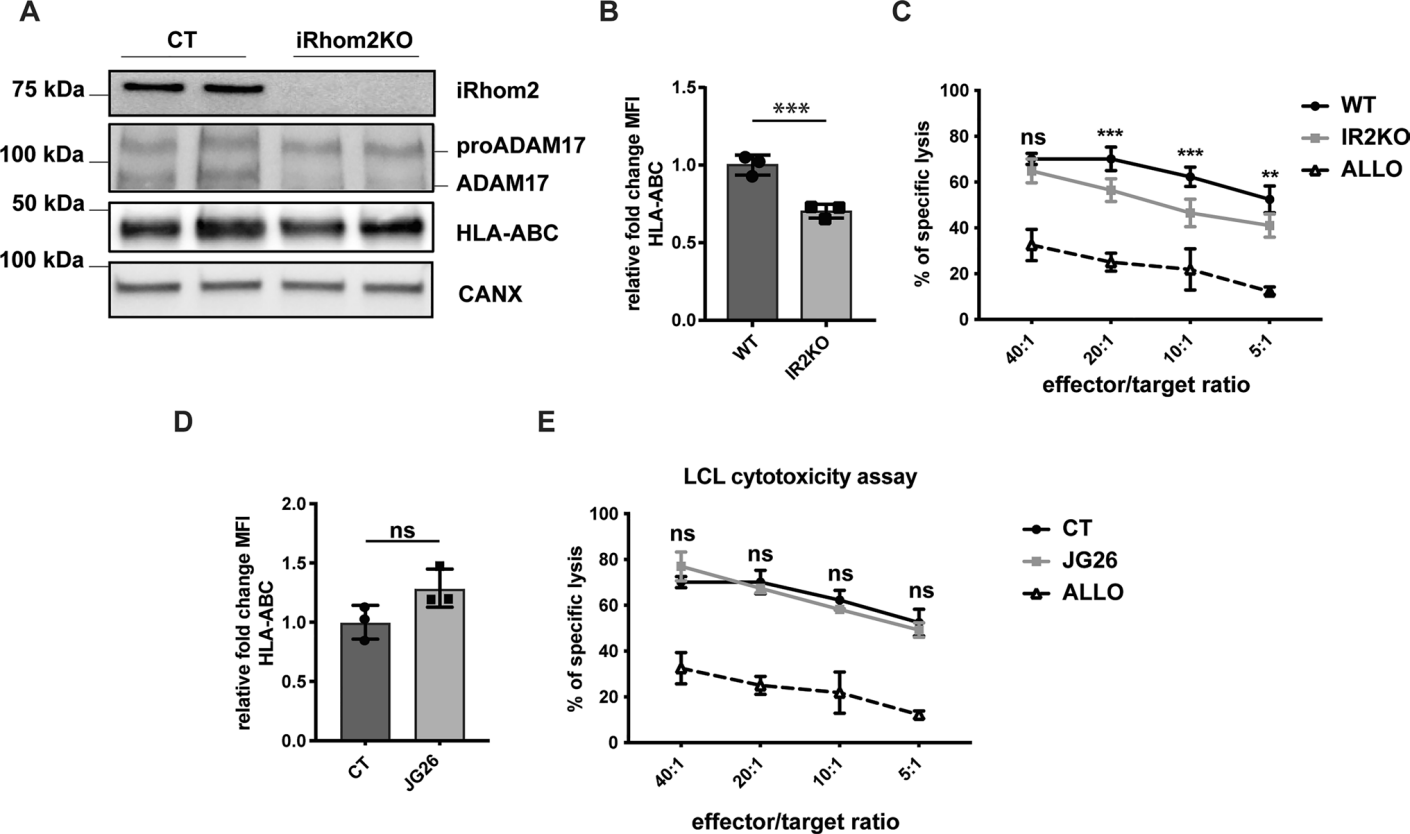
iRhom2 regulates activation of CD8 + T cells by EBV-infected LCL. **A** Western blots showing levels of iRhom2, ADAM17, HLA and calnexin (CANX) in the lysate of an iRhom2 KO lymphoblastoid cell line (LCL) compared to its wild-type counterpart. **B** Cell membrane levels of HLA in iRhom2 KO LCL and its wild-type controls measured as the mean fluorescent intensity (MFI) by flow cytometry (***p < 0.005; Student’s t-test). **C** Comparison of cytotoxicity obtained at 4 h by the chromium release assay at specific ratios of CD8 + T cells and iRhom2 KO or WT LCL. Values are represented as % of released versus the total incorporated 51Cr. Six biological replicates were analysed and results were statistically evaluated by the 2-way ANOVA, for each T cell/LCL ratio. In addition, results were further analysed by the Tukey’s multiple comparison test, which compares all pairs of iRhom2 and WT means and used to find whether means are significantly different from each other (Predicted least squares mean difference: 11.51; 95% of confidence interval of difference: 7,476 to 15,55; adjusted *p* value < 0.001). WT LCL were incubated with allogenic T cells as a negative control. **D** Cell membrane levels of HLA in JG26-treated LCL and untreated controls (CT) measured as the mean fluorescent intensity (MFI) by flow cytometry and statistically analysed by Student’s t-test (*p* value > 0.05). **E** Comparison of cytotoxicity obtained at 4 h by the chromium release assay at specific ratios of CD8 + T cells and LCL, in the presence or absence of 5 µM JG26. Values are represented as % of released versus the total incorporated ^51^Cr. Three biological replicates were analysed and results were statistically evaluated by the 2-way ANOVA). Of note, the control experiment in which LCLs were incubated with allogenic CTLs, displayed in C and E as open triangles and a black dashed line, is the same

Finally, we planned to investigate whether reduced membrane levels of MHC-I, as a consequence of iRhom2 ablation, could impair cytotoxic CD8 + T cells (CTLs) activation. Thus, we used an Epstein–Barr virus (EBV)-transformed lymphoblastoid cell line (LCL), obtained by infecting peripheral blood monocular cells in vitro [28]. Epstein-Barr virus is a herpes-virus that is endemic in the human population. Infection of B cells with EBV leads to development of an immortalized LCL that has been widely used to investigate virus-mediated immune responses [18, 39]. When an EBV-transformed LCL is incubated with CTLs isolated and expanded from the same individual, CTLs will recognize the viral antigen bound to MHC-I on LCL through their T cell receptor (TCR), and induce cell-mediated response against infected LCL cells, culminating in their lysis. In order to investigate whether loss of iRhom2 and subsequent reduction of surface levels of MHC-I could impair CTL activation, iRhom2 was ablated in a LCL (which led to impairment of ADAM17 maturation and reduction of HLA on the cell surface—Fig. 6A and B), labelled with ^51^Cr and incubated with autologous CTLs (isolated from the same individual–Supplemental Fig. 5).

Cell mediated cytotoxicity was significantly reduced in iR2KO LCL compared to WT controls at 20:1, 10:1 and 5:1 ratio. At 40:1 ratio, which is much higher than the physiological effector\target ratio, CTLs induced a similar response against iR2KO and WT LCL (Fig. 5C). Due to MHC-I restriction, LCL induced a much lower lytic response when it was incubated with allogenic CTLs, further proving the specificity of the MHC-I/CTL interaction in this model. Differently from iRhom2 loss, ADAM17 inhibition by JG26 did not alter cell surface levels of HLA on LCL (Fig. 6D), and LCL ability to induce CTL response (Fig. 6E). These results suggest that iRhom2 can affect CTL-mediated responses through its ability to control cell surface bioavailability of MHC-I rather than ADAM17-mediated shedding.

## Discussion

In the last decade, iRhoms have emerged as essential regulators of ADAM17, in that they can control trafficking, maturation and, ultimately, activity of the proteinase [6]. iRhom1 and iRhom2 are both expressed in most of mammalian tissues, where they have some redundancy with respect to their ability to support the maturation of ADAM17. The immune system represents an interesting exception to this common theme, as only iRhom2, but not iRhom1, is expressed [7, 8, 12]. As a consequence, shedding of ADAM17 substrates and regulation of their related molecular pathways, e.g. TNF release and initiation of immune responses, strictly depend on iRhom2 [9]. In order to identify additional substrates of iRhom2/ADAM17 in macrophages, and therefore uncover new functions of iRhom2 in immunity, we applied unbiased secretome analysis to primary macrophages isolated from iRhom2 KO mice that, differently from ADAM17 KO mice, do not die perinatally. We stimulated iRhom2 KO murine macrophages with different amounts of LPS, either alone or with PMA, as we reasoned that combined stimulation with LPS and PMA could have synergistic effects on ADAM17 activation and lead to a broader identification of its substrates. We found that extracellular levels of H2-D1 (a murine haplotype of MHC-I molecules) were consistently diminished in all different datasets. We validated this mechanism by orthogonal methods in human, where MHC-I molecules are known as the human leukocyte antigen (HLA) system. Interestingly, we found that the decrease of soluble MHC-I molecules in the secretome of iRhom2-deficient macrophages was not only due to its impaired ADAM17-dependent proteolytic release, but also to their diminished expression on the cell surface. This dual mechanism was evident in cells with a myeloid origin, such as macrophage-like (THP-1) cells, and fibroblasts where both iRhom1 and iRhom2 are present. Mechanistically, how iRhom2 controls surface levels of MHC-I molecules has to be further elucidated. We reasoned that MHC-I molecules could be iRhom2 clients, additional to ADAM17 [9, 10]. However, we could not predict a direct interaction between iRhom2 and MHC-I by using computational modelling. In agreement, three separate groups performed a mass spectrometry-based screening of proteins interacting with iRhom2 and did not identify MHC-I molecules as putative interactors [40–42]. However, these findings do not rule out an interaction of MHC-I molecules with the iRhom2/ADAM17 complex. We also observed that the kinetics of iRhom2 control of ADAM17 or MHC-I trafficking are clearly different. While ADAM17 gets trapped in the ER when iRhom2 is ablated, and, subsequently, no mature ADAM17 is found at the cell membrane of iRhom2 KO macrophages, iRhom2 loss does not fully abolish MHC-I trafficking to the cell membrane. We found that in the absence of iRhom2 membrane levels of MHC-I diminished between 20 and 50%, depending on cell type. These results suggest that, rather than acting as an essential cofactor, as it is for ADAM17, iRhom2 might facilitate one or more steps of the MHC-I antigen presentation pathway. Antigens destined to be loaded onto MHC-I molecules arise from viral proteins or mutated proteins, such as oncogenes [43]. These 8–10 amino-acid long peptides are generated in the cytoplasm through proteasomal degradation and then translocated into the ER, where the peptide loading complex (PLC) ensures the MHC class I:peptide assembly [44]. The PLC comprises seven different subunits that are precisely coordinated in a single macromolecular complex: the transporter associated with antigen processing (which includes two subunits, TAP1 and TAP2, jointly referred to as TAP), the oxidoreductase ERp57, the nascent MHC-I molecule (a heterodimer consisting of the α chain bound to the β-microglobulin) and the chaperones tapasin and calreticulin [44]. Antigens are translocated from the cytosol into the ER by TAP [45]. Nascent MHC-I molecules are highly unstable and the PLC ensures their stabilization until the peptide antigen is correctly loaded into the antigen-biding cleft. Once MHC-I molecules are correctly loaded with the antigen peptides, they can leave the ER and reach the plasma membrane through the secretory pathway [43, 44]. We can speculate that iRhom2 plays a role in this process and, based on what we currently know about its functions, it can occur through distinct mechanisms. For instance, iRhom2 could regulate the stability of PLC or other proteins involved in the MHC-I antigen presentation pathway, in a similar manner to its regulation of ubiquitination and therefore stability of STING and VISA [15, 16] Indeed, iRhom2 prevents proteasomal degradation of STING by recruiting the deubiquitinase EIF3S5 that removes K48-linked polyubiquitin chains from the protein. Similarly, iRhom2 inhibits the auto-ubiquitination and degradation of the E3 ubiquitin ligase RNF5, of which VISA is a target, therefore antagonizing its proteasomal degradation [16]. Given that the stability of MHC-I is not directly regulated by iRhom2, as its levels were not diminished in iRhom2-deficient cells, one possibility is that iRhom2 could regulate the stability of proteins involved in the MHC-I antigen presentation pathway. In line, ablation of PLC subunits, including TAP1 and tapasin, reduced surface presentation of MHC-I molecules to a similar extent than iRhom2 loss [46]. Another possibility is that iRhom2 could facilitate ER-exit of MHC-I molecules. While key steps of MHC-I assembly, peptide-loading and release of loaded MHC-I from the PLC are relatively well defined, little is known about how MHC-I molecules exit the ER en route to the cell membrane, and whether this occurs by bulk flow or through a selective receptor-mediated mechanism. However, the similar levels of endo-H resistant HLA in WT and iRhom2KO cells argues against this interpretation. Instead, our results suggest that iRhom2/ADAM17 have a role in regulating the levels of HLA at the cell surface, either by increasing transport from the Golgi to the cell surface or by stabilizing the protein at the cell surface, thereby reducing endocytosis and degradation. It is also possible that iRhom2/ADAM17 cleave and shed or inactivate another protease that can process HLA on the cell surface, which would accumulate and be more active in the absence of iRhom2/ADAM17. Further studies will be necessary to distinguish between these possibilities.

In addition to MHC-I molecules, our mass spectrometry-based analysis identified a heterogenous collection of proteins that are shed by activated ADAM17 in murine and human macrophages, and suggest that the iRhom2/ADAM17 proteolytic complex has a broader function in immunity than currently appreciated. In addition to the known ADAM17 substrates CSF1R and AXL, mass-spectrometry pinpointed a number of putative novel ADAM17 substrates in murine macrophages. Among them, the interleukin-1 receptor accessory protein (IL1RAP) emerged as regulated by iRhom2/ADAM17 in different proteomic datasets. IL1RAP is also known to play an essential role in the signaling of the IL-1 family cytokines such as IL-1, IL-33 and IL-36 [47]. This suggests that iRhom2/ADAM17 could control other pro-inflammatory pathways, in addition to TNF and IL-6. Similarly, mass spectrometry indicated that release of the signal regulatory protein α (SIRPα) in monocyte-derived macrophages diminished when iRhom2 was ablated, in line with a previous report describing its ADAM-dependent shedding in stimulated neurons [48]. SIRPα functions as inhibitory receptor that sends a “don’t eat me” signal to the effector cells of the innate immune systems. Interestingly, this “don’t eat me” signal is akin to the one provided by MHC class I molecules to NK cells via Ig-like or Ly49 receptors, suggesting that iRhom2/ADAM17 could play an unprecedented wide role in immune recognition [49].

In conclusion, by using an unbiased high-resolution proteomic approach, we found that iRhom2 controls another key player in immunity, which is MHC-I. This occurs by a dual mechanism. On the one hand, iRhom2 controls levels of MHC-I at the cell surface and the capability of target cells to display viral antigens for T cell recognition. On the other, iRhom2 can support the stimulated ADAM17-dependent shedding of MHC-I. The peculiar expression pattern of mouse iRhom2, which is present in all tissues, especially high in the immune cells, and has low, if any expression in neurons, mainly overlaps with that of MHC-I molecules that are expressed in all nucleated cells and confined to microglial cells within the nervous system [50, 51]. This expression overlap, in line with our findings, suggests that the biological role of iRhom2 and MHC-I molecules can be strongly associated in vivo.

### Supplementary Information

Below is the link to the electronic supplementary material.

Supplementary file1 Supplemental Figure 1 (A) Number of proteins annotated as secreted or membrane proteins out of 380 proteins in total detected in the secretome of unstimulated BMDMs and 490 proteins in total detected in the secretome of stimulated BMDMs. The number of membrane proteins was further divided by topology in multipass, type I transmembrane (TM1), type II transmembrane (TM2), GPI-anchored (GPI) proteins and proteins with a different topology (other membrane). (B) Levels of TNFα released by iRhom2 KO or WT BMDMs, stimulated with 100 ng/ml LPS or 100 ng/ml LPS and 25 ng/ml PMA for 1 h, measured by mass-spectrometry and subjected to data-dependent acquisition and label-free quantification. (C) QARIP analysis of the transmembrane proteins that were found significantly reduced in the secretome of LPS/PMA stimulated iRhom2 KO BMDMs. (D-F) Volcano plot showing the -Log10 of p-values versus the log2 of protein ratio between iRhom2KO and WT BMDMs stimulated with 100 ng/ml LPS for 1 h (D), iRhom2KO and WT BMDMs stimulated with 100 ng/ml LPS and 25 ng/ml PMA for 6 h (E) and iRhom2KO and WT BMDMs stimulated with 1 µg/ml LPS for 1 h (F). (G-H) Flow cytometry analysis showing cell surface levels of H2-D1 in TAPI-treated and control BMDMs, either under steady-state conditions (G) or upon LPS/PMA stimulation (H). (TIF 35754 KB)

Supplementary file2 Supplemental Figure 2 (A) Expression levels of iRhom2 (aka RHBDF2 by gene name) in iRhom2 knockdown PBMC-derived macrophages and their controls measured by quantitative qPCR. (B) Volcano plot showing the -Log10 of p-values versus the log2 of protein ratio between LPS/PMA stimulated and control THP-1 cells. The horizontal line indicates the –log10(p-value) of 1.3, which corresponds to a p-value of 0.05. Proteins above this line are considered significantly regulated. Transmembrane proteins significantly increased upon stimulation are displayed as red filled dots (putative ADAM17 substrates fall within this group), other proteins as gray dots. (C) Immunoblots showing the levels of shed HLA in the conditioned media, and full-length HLA in the cell lysates of THP-1 cells treated or not with 100 ng/ml LPS and 25 ng/ml PMA for 3 h. Calnexin is used as a protein loading control. (D) Densitometric quantification of shed HLA in the conditioned media of THP-1 treated or not with LPS/PMA. (E) Immunoblots showing ADAM17 maturation in iRhom2 knockdown and control THP-1 cells (treated with RHBDF2 or non-targeting siRNAs, respectively); and mRNA expression of iRhom2 (RHBDF2 by gene name) in iRhom2 knockdown THP-1 cells and controls measured by qPCR. (F) Immunoblots and qPCR showing that protein and mRNA levels of ADAM17 in THP-1 cells decreased upon its silencing with specific siRNAs (siADAM17) compared to controls treated with non-targeting (NT) siRNAs. (G-H) Immunoblots (G) and their relative quantifications (H) showing levels of shed HLA in the conditioned media, and full length HLA in the lysates of control (CT) and ADAM17 knockdown THP-1 cells. (I) Immunoblots showing levels of ADAM17 (and calnexin – CANX) in wild-type and ADAM17 knockout THP-1 cells. (J) Volcano plot showing the -Log10 of p-values versus the log2 of protein ratio between LPS/PMA stimulated THP-1 cells, in the presence or absence of JG26. The horizontal line indicates the –log10(p-value) of 1.3, which corresponds to a p-value of 0.05. Proteins above this line are considered significantly regulated. Transmembrane proteins significantly increased in the absence of the ADAM17 inhibitor are displayed as red filled dots, other proteins as grey filled dots. (TIF 35824 KB)

Supplementary file3 Supplemental Figure 3 (A) Levels of shed H2-D1 in the conditioned media of iRhom1 KO, iRhom2 KO, iRhom1/2 double KO MEFs or ADAM17 KO MEFs, stimulated or not with PMA, measured by mass-spectrometry and label-free quantification. (**p < 0.01, ns non-significant; Student’s t-test). (B) mRNA expression of H2-D1, iRhom1 and iRhom2, measured by qPCR, in iRhom1 KO, iRhom2 KO, iRhom1/2 double KO and ADAM17 KO MEFs. (C) Immunoblots showing levels of iRhom2, ADAM17 and calnexin in HTB94 cells, where iRhom2 or ADAM17 were ablated through CRISPR-Cas9 (parental: untreated HTB94 cells; NTC: HTB94 cells treated with non-targeting guide RNA). (D) Flow cytometry analysis of HLA levels in iRhom2 KO, ADAM17 KO and WT HTB94 cells. (E). Immunoblots showing HLA in lysates of iRhom2 KO or WT HTB94 cells treated with endoglycosidase H (Endo H), Peptide-N-Glycosidase F (PNGaseF) or control buffer (CT). (F) Immunoblots showing HLA in lysates of iRhom2 KO or WT THP1 cells treated with endoglycosidase H (Endo H), Peptide-N-Glycosidase F (PNGaseF) or control buffer (CT). (TIF 36198 KB)

Supplementary file4 Supplemental Figure 4 (A) Structural modelling of iRhom2 (in red) with HLA-A (A), ADAM17 (B) or ADAM10 (C) (in blue), using the deep learning algorithm AlphaFold 2. Conversely to the iRhom2/ADAM17 predicted interaction, the predicted align error (PAE) is low for an iRhom2/HLA-A interaction, as well as for an iRhom2/ADAM10 interaction, and therefore not supporting an interaction between iRhom2 and HLA-A. (TIF 38570 KB)

Supplementary file5 (XLSX 304 KB)

Supplementary file6 (XLSX 99 KB)

Supplementary file7 (XLSX 1046 KB)

## Data Availability

Data will be made available on reasonable request.

## References

[1] Black RA (1997). A metalloproteinase disintegrin that releases tumour-necrosis factor-alpha from cells. Nature.

[2] Calligaris M (2021). Strategies to target ADAM17 in disease: from its discovery to the iRhom revolution. Molecules.

[3] Moss ML (1997). Cloning of a disintegrin metalloproteinase that processes precursor tumour-necrosis factor-alpha. Nature.

[4] Peschon JJ (1998). An essential role for ectodomain shedding in mammalian development. Science.

[5] Zunke F, Rose-John S (2017). The shedding protease ADAM17: physiology and pathophysiology. Biochim Biophys Acta Mol Cell Res.

[6] Dulloo I, Muliyil S, Freeman M (2019). The molecular, cellular and pathophysiological roles of iRhom pseudoproteases. Open Biol.

[7] Christova Y, Adrain C, Bambrough P, Ibrahim A, Freeman M (2013). Mammalian iRhoms have distinct physiological functions including an essential role in TACE regulation. EMBO Rep.

[8] Li X (2015). iRhoms 1 and 2 are essential upstream regulators of ADAM17-dependent EGFR signaling. Proc Natl Acad Sci U S A.

[9] McIlwain DR (2012). iRhom2 regulation of TACE controls TNF-mediated protection against listeria and responses to LPS. Science.

[10] Adrain C, Zettl M, Christova Y, Taylor N, Freeman M (2012). Tumor necrosis factor signaling requires iRhom2 to promote trafficking and activation of TACE. Science.

[11] Giese AA (2021). Inflammatory activation of surface molecule shedding by upregulation of the pseudoprotease iRhom2 in colon epithelial cells. Sci Rep.

[12] Issuree PD (2013). iRHOM2 is a critical pathogenic mediator of inflammatory arthritis. J Clin Invest.

[13] Zhao Y (2022). Identification of molecular determinants in iRhoms1 and 2 that contribute to the substrate selectivity of stimulated ADAM17. Int J Mol Sci.

[14] Maretzky T (2013). iRhom2 controls the substrate selectivity of stimulated ADAM17-dependent ectodomain shedding. Proc Natl Acad Sci U S A.

[15] Luo WW (2016). iRhom2 is essential for innate immunity to DNA viruses by mediating trafficking and stability of the adaptor STING. Nat Immunol.

[16] Luo WW (2017). iRhom2 is essential for innate immunity to RNA virus by antagonizing ER- and mitochondria-associated degradation of VISA. PLoS Pathog.

[17] Meissner F, Scheltema RA, Mollenkopf HJ, Mann M (2013). Direct proteomic quantification of the secretome of activated immune cells. Science.

[18] Khanolkar A (2003). CD4+ T cell-induced differentiation of EBV-transformed lymphoblastoid cells is associated with diminished recognition by EBV-specific CD8+ cytotoxic T cells. J Immunol.

[19] Weiss M, Blazek K, Byrne AJ, Perocheau DP, Udalova IA (2013). IRF5 is a specific marker of inflammatory macrophages in vivo. Mediators Inflamm.

[20] Wisniewski JR, Zougman A, Nagaraj N, Mann M (2009). Universal sample preparation method for proteome analysis. Nat Methods.

[21] Rappsilber J, Ishihama Y, Mann M (2003). Stop and go extraction tips for matrix-assisted laser desorption/ionization, nanoelectrospray, and LC/MS sample pretreatment in proteomics. Anal Chem.

[22] Ivankov DN (2013). QARIP: a web server for quantitative proteomic analysis of regulated intramembrane proteolysis. Nucleic Acids Res.

[23] Tarique AA (2015). Phenotypic, functional, and plasticity features of classical and alternatively activated human macrophages. Am J Respir Cell Mol Biol.

[24] Schwende H, Fitzke E, Ambs P, Dieter P (1996). Differences in the state of differentiation of THP-1 cells induced by phorbol ester and 1,25-dihydroxyvitamin D3. J Leukoc Biol.

[25] Nuti E (2013). Selective arylsulfonamide inhibitors of ADAM-17: hit optimization and activity in ovarian cancer cell models. J Med Chem.

[26] Weskamp G (2020). ADAM17 stabilizes its interacting partner inactive rhomboid 2 (iRhom2) but not inactive rhomboid 1 (iRhom1). J Biol Chem.

[27] Jocher G (2022). ADAM10 and ADAM17 promote SARS-CoV-2 cell entry and spike protein-mediated lung cell fusion. EMBO Rep.

[28] Neitzel H (1986). A routine method for the establishment of permanent growing lymphoblastoid cell lines. Hum Genet.

[29] Kahveci-Turkoz S (2023). A structural model of the iRhom-ADAM17 sheddase complex reveals functional insights into its trafficking and activity. Cell Mol Life Sci.

[30] Horiuchi K (2007). Substrate selectivity of epidermal growth factor-receptor ligand sheddases and their regulation by phorbol esters and calcium influx. Mol Biol Cell.

[31] Le Gall SM (2010). ADAM17 is regulated by a rapid and reversible mechanism that controls access to its catalytic site. J Cell Sci.

[32] Lichtenthaler SF, Lemberg MK, Fluhrer R (2018). Proteolytic ectodomain shedding of membrane proteins in mammals-hardware, concepts, and recent developments. EMBO J.

[33] Qing X (2016). iRhom2 regulates CSF1R cell surface expression and non-steady state myelopoiesis in mice. Eur J Immunol.

[34] Orme JJ (2016). Heightened cleavage of Axl receptor tyrosine kinase by ADAM metalloproteases may contribute to disease pathogenesis in SLE. Clin Immunol.

[35] Zubarev RA (2013). The challenge of the proteome dynamic range and its implications for in-depth proteomics. Proteomics.

[36] Schumacher N (2023). EGFR stimulation enables IL-6 trans-signalling via iRhom2-dependent ADAM17 activation in mammary epithelial cells. Biochim Biophys Acta Mol Cell Res.

[37] Vincent B (2001). The disintegrins ADAM10 and TACE contribute to the constitutive and phorbol ester-regulated normal cleavage of the cellular prion protein. J Biol Chem.

[38] Malapeira J, Esselens C, Bech-Serra JJ, Canals F, Arribas J (2011). ADAM17 (TACE) regulates TGFbeta signaling through the cleavage of vasorin. Oncogene.

[39] Miller G, Lipman M (1973). Release of infectious epstein-barr virus by transformed marmoset leukocytes. Proc Natl Acad Sci U S A.

[40] Kunzel U (2018). FRMD8 promotes inflammatory and growth factor signalling by stabilising the iRhom/ADAM17 sheddase complex. Elife.

[41] Oikonomidi I (2018). iTAP, a novel iRhom interactor, controls TNF secretion by policing the stability of iRhom/TACE. Elife.

[42] Dusterhoft S (2021). The iRhom homology domain is indispensable for ADAM17-mediated TNFalpha and EGF receptor ligand release. Cell Mol Life Sci.

[43] Hewitt EW (2003). The MHC class I antigen presentation pathway: strategies for viral immune evasion. Immunology.

[44] Blees A (2017). Structure of the human MHC-I peptide-loading complex. Nature.

[45] Ritz U, Seliger B (2001). The transporter associated with antigen processing (TAP): structural integrity, expression, function, and its clinical relevance. Mol Med.

[46] Barends M (2023). Dynamic interactome of the MHC I peptide loading complex in human dendritic cells. Proc Natl Acad Sci U S A.

[47] Zarezadeh Mehrabadi A (2022). The roles of interleukin-1 receptor accessory protein in certain inflammatory conditions. Immunology.

[48] Toth AB (2013). Synapse maturation by activity-dependent ectodomain shedding of SIRPalpha. Nat Neurosci.

[49] Barclay AN (2009). Signal regulatory protein alpha (SIRPalpha)/CD47 interaction and function. Curr Opin Immunol.

[50] Lichtenthaler SF, O’Hara BF, Blobel CP (2015). iRhoms in the brain-a new frontier?. Cell Cycle.

[51] Wong GH, Bartlett PF, Clark-Lewis I, Battye F, Schrader JW (1984). Inducible expression of H-2 and Ia antigens on brain cells. Nature.

