# Supplementary figures and images for: iRhom2 regulates ectodomain shedding and surface expression of the major histocompatibility complex (MHC) class I
Source: Cell Mol Life Sci. 2024 Apr 4;81(1):163. doi: 10.1007/s00018-024-05201-7 (PMC10991058; doi:10.1007/s00018-024-05201-7)

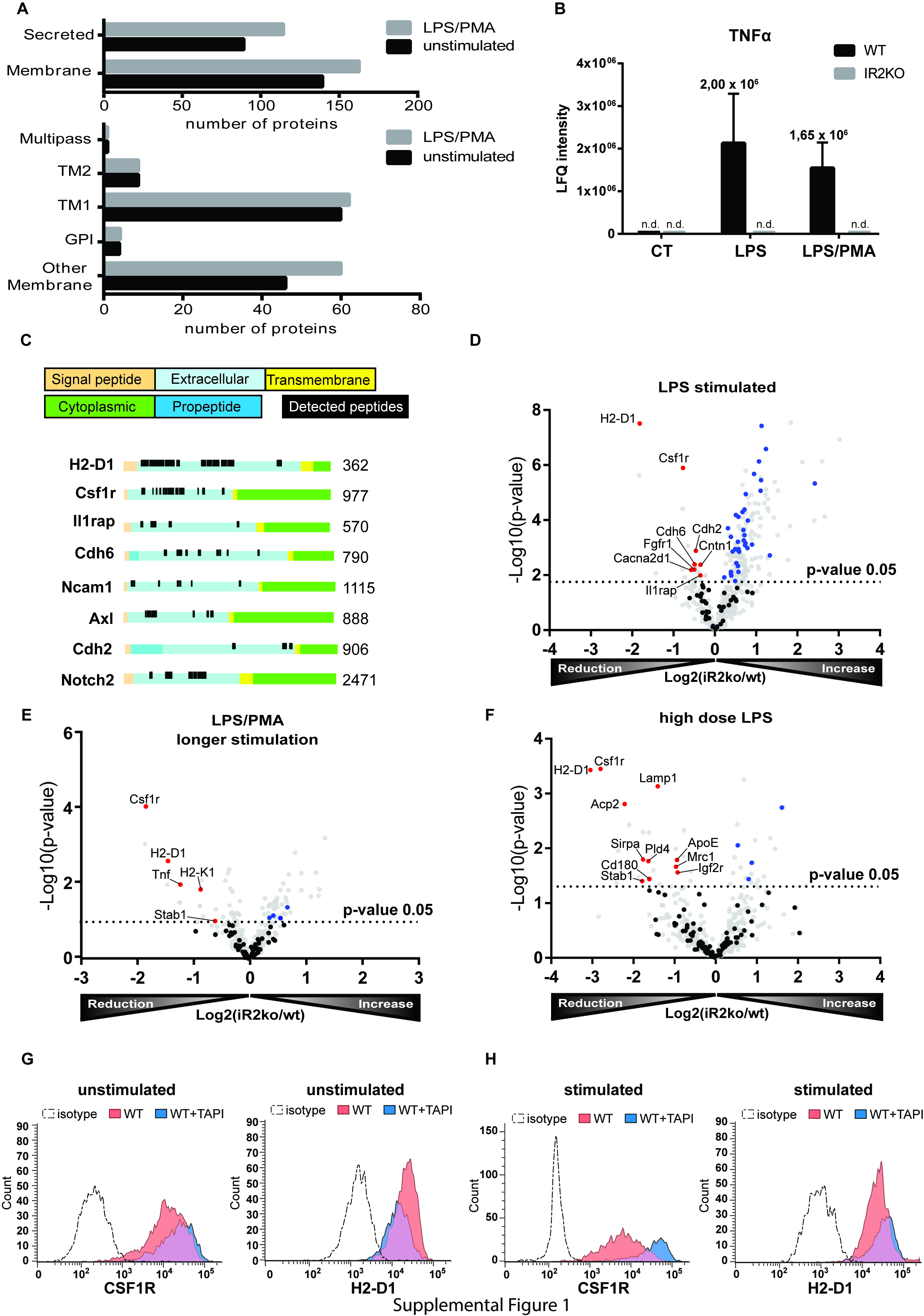

Supplement: Supplementary file 1 — Supplementary file1 Supplemental Figure 1 (A) Number of proteins annotated as secreted or membrane proteins out of 380 proteins in total detected in the secretome of unstimulated BMDMs and 490 proteins in total detected in the secretome of stimulated BMDMs. The number of membrane proteins was further divided by topology in multipass, type I transmembrane (TM1), type II transmembrane (TM2), GPI-anchored (GPI) proteins and proteins with a different topology (other membrane). (B) Levels of TNFα released by iRhom2 KO or WT BMDMs, stimulated with 100 ng/ml LPS or 100 ng/ml LPS and 25 ng/ml PMA for 1 h, measured by mass-spectrometry and subjected to data-dependent acquisition and label-free quantification. (C) QARIP analysis of the transmembrane proteins that were found significantly reduced in the secretome of LPS/PMA stimulated iRhom2 KO BMDMs. (D-F) Volcano plot showing the -Log10 of p-values versus the log2 of protein ratio between iRhom2KO and WT BMDMs stimulated with 100 ng/ml LPS for 1 h (D), iRhom2KO and WT BMDMs stimulated with 100 ng/ml LPS and 25 ng/ml PMA for 6 h (E) and iRhom2KO and WT BMDMs stimulated with 1 µg/ml LPS for 1 h (F). (G-H) Flow cytometry analysis showing cell surface levels of H2-D1 in TAPI-treated and control BMDMs, either under steady-state conditions (G) or upon LPS/PMA stimulation (H). (TIF 35754 KB) [file 18_2024_5201_MOESM1_ESM.tif]

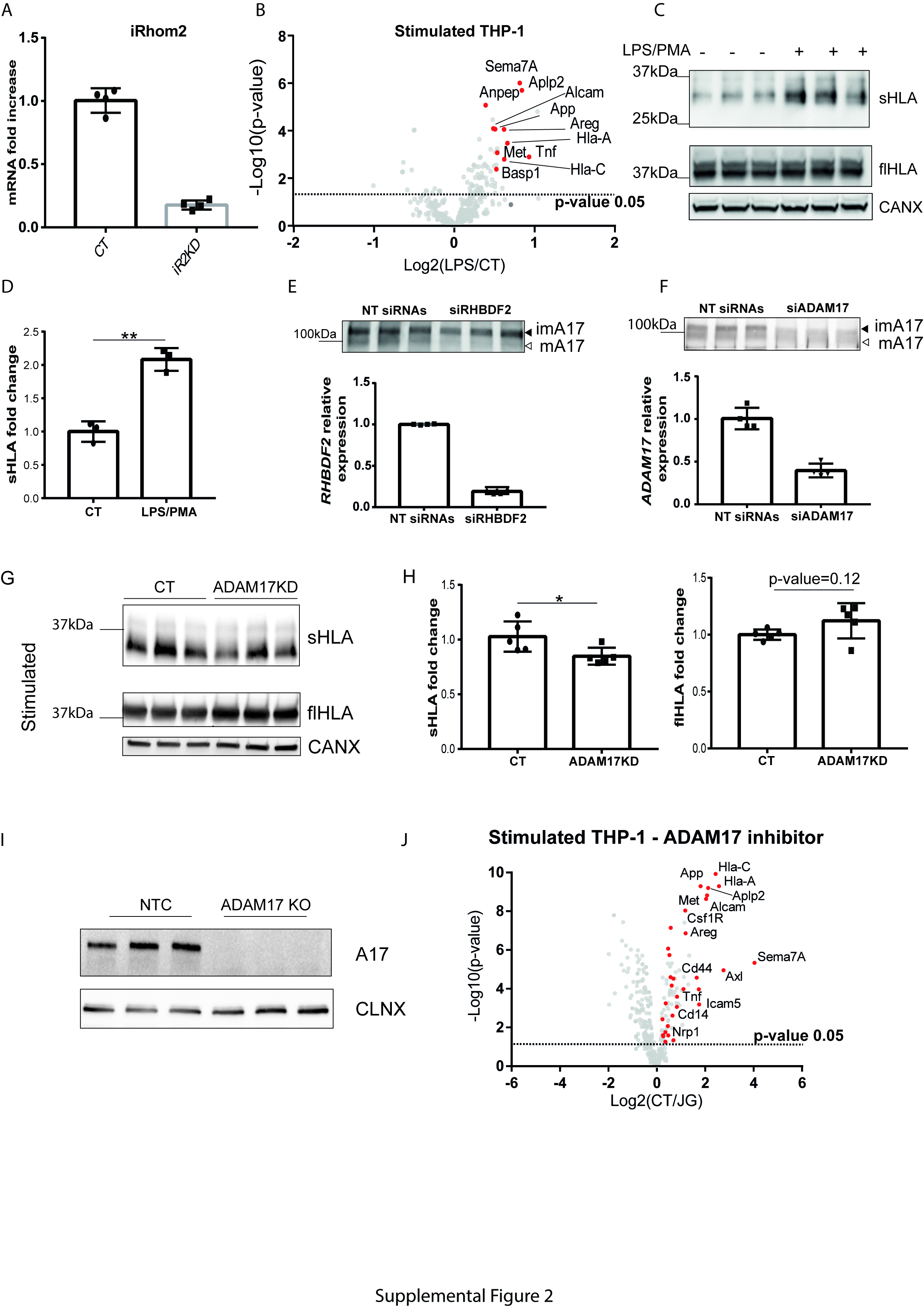

Supplement: Supplementary file 2 — Supplementary file2 Supplemental Figure 2 (A) Expression levels of iRhom2 (aka RHBDF2 by gene name) in iRhom2 knockdown PBMC-derived macrophages and their controls measured by quantitative qPCR. (B) Volcano plot showing the -Log10 of p-values versus the log2 of protein ratio between LPS/PMA stimulated and control THP-1 cells. The horizontal line indicates the –log10(p-value) of 1.3, which corresponds to a p-value of 0.05. Proteins above this line are considered significantly regulated. Transmembrane proteins significantly increased upon stimulation are displayed as red filled dots (putative ADAM17 substrates fall within this group), other proteins as gray dots. (C) Immunoblots showing the levels of shed HLA in the conditioned media, and full-length HLA in the cell lysates of THP-1 cells treated or not with 100 ng/ml LPS and 25 ng/ml PMA for 3 h. Calnexin is used as a protein loading control. (D) Densitometric quantification of shed HLA in the conditioned media of THP-1 treated or not with LPS/PMA. (E) Immunoblots showing ADAM17 maturation in iRhom2 knockdown and control THP-1 cells (treated with RHBDF2 or non-targeting siRNAs, respectively); and mRNA expression of iRhom2 (RHBDF2 by gene name) in iRhom2 knockdown THP-1 cells and controls measured by qPCR. (F) Immunoblots and qPCR showing that protein and mRNA levels of ADAM17 in THP-1 cells decreased upon its silencing with specific siRNAs (siADAM17) compared to controls treated with non-targeting (NT) siRNAs. (G-H) Immunoblots (G) and their relative quantifications (H) showing levels of shed HLA in the conditioned media, and full length HLA in the lysates of control (CT) and ADAM17 knockdown THP-1 cells. (I) Immunoblots showing levels of ADAM17 (and calnexin – CANX) in wild-type and ADAM17 knockout THP-1 cells. (J) Volcano plot showing the -Log10 of p-values versus the log2 of protein ratio between LPS/PMA stimulated THP-1 cells, in the presence or absence of JG26. The horizontal line indicates the –log10(p-value) o [file 18_2024_5201_MOESM2_ESM.tif]

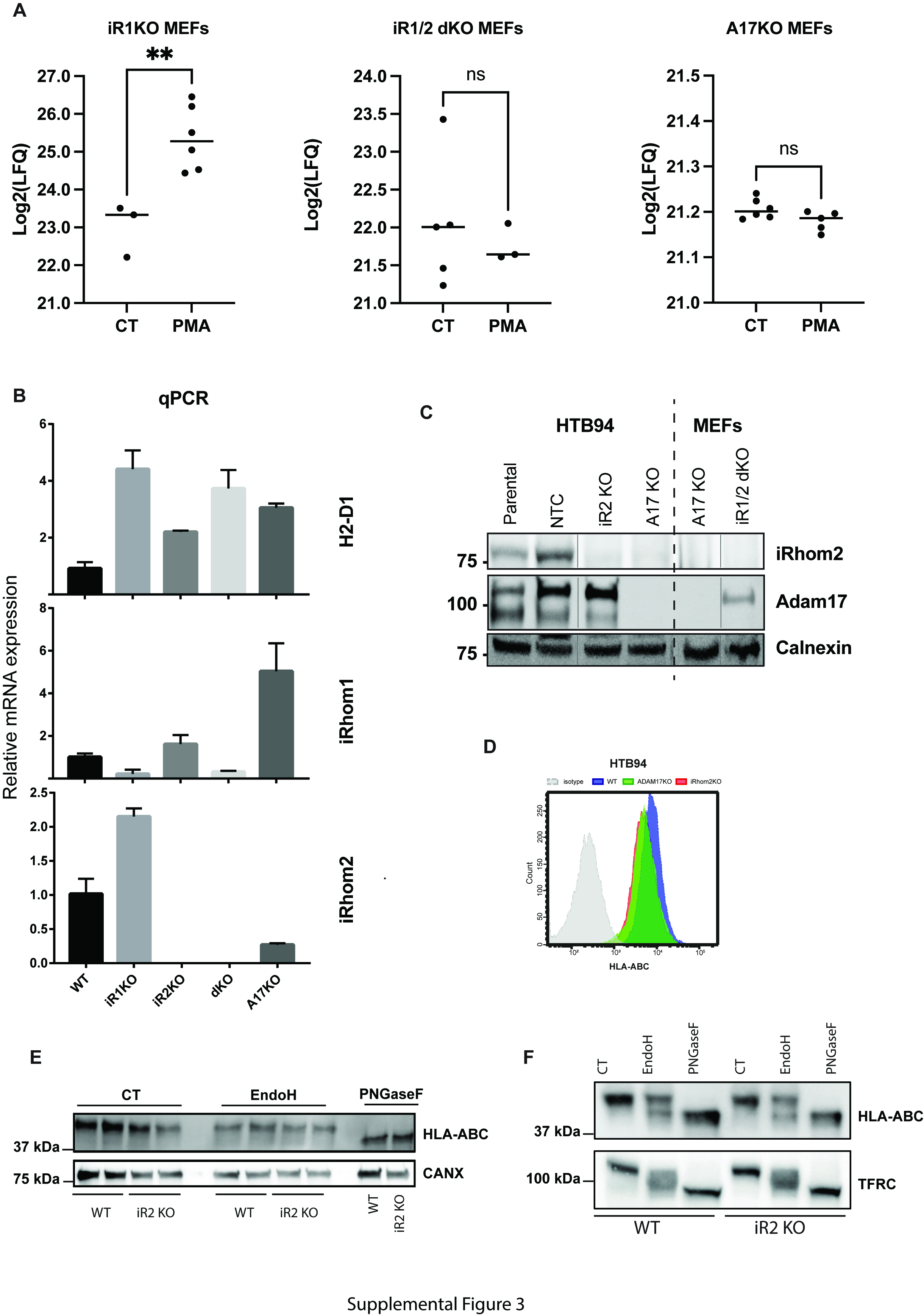

Supplement: Supplementary file 3 — Supplementary file3 Supplemental Figure 3 (A) Levels of shed H2-D1 in the conditioned media of iRhom1 KO, iRhom2 KO, iRhom1/2 double KO MEFs or ADAM17 KO MEFs, stimulated or not with PMA, measured by mass-spectrometry and label-free quantification. (**p < 0.01, ns non-significant; Student’s t-test). (B) mRNA expression of H2-D1, iRhom1 and iRhom2, measured by qPCR, in iRhom1 KO, iRhom2 KO, iRhom1/2 double KO and ADAM17 KO MEFs. (C) Immunoblots showing levels of iRhom2, ADAM17 and calnexin in HTB94 cells, where iRhom2 or ADAM17 were ablated through CRISPR-Cas9 (parental: untreated HTB94 cells; NTC: HTB94 cells treated with non-targeting guide RNA). (D) Flow cytometry analysis of HLA levels in iRhom2 KO, ADAM17 KO and WT HTB94 cells. (E). Immunoblots showing HLA in lysates of iRhom2 KO or WT HTB94 cells treated with endoglycosidase H (Endo H), Peptide-N-Glycosidase F (PNGaseF) or control buffer (CT). (F) Immunoblots showing HLA in lysates of iRhom2 KO or WT THP1 cells treated with endoglycosidase H (Endo H), Peptide-N-Glycosidase F (PNGaseF) or control buffer (CT). (TIF 36198 KB) [file 18_2024_5201_MOESM3_ESM.tif]

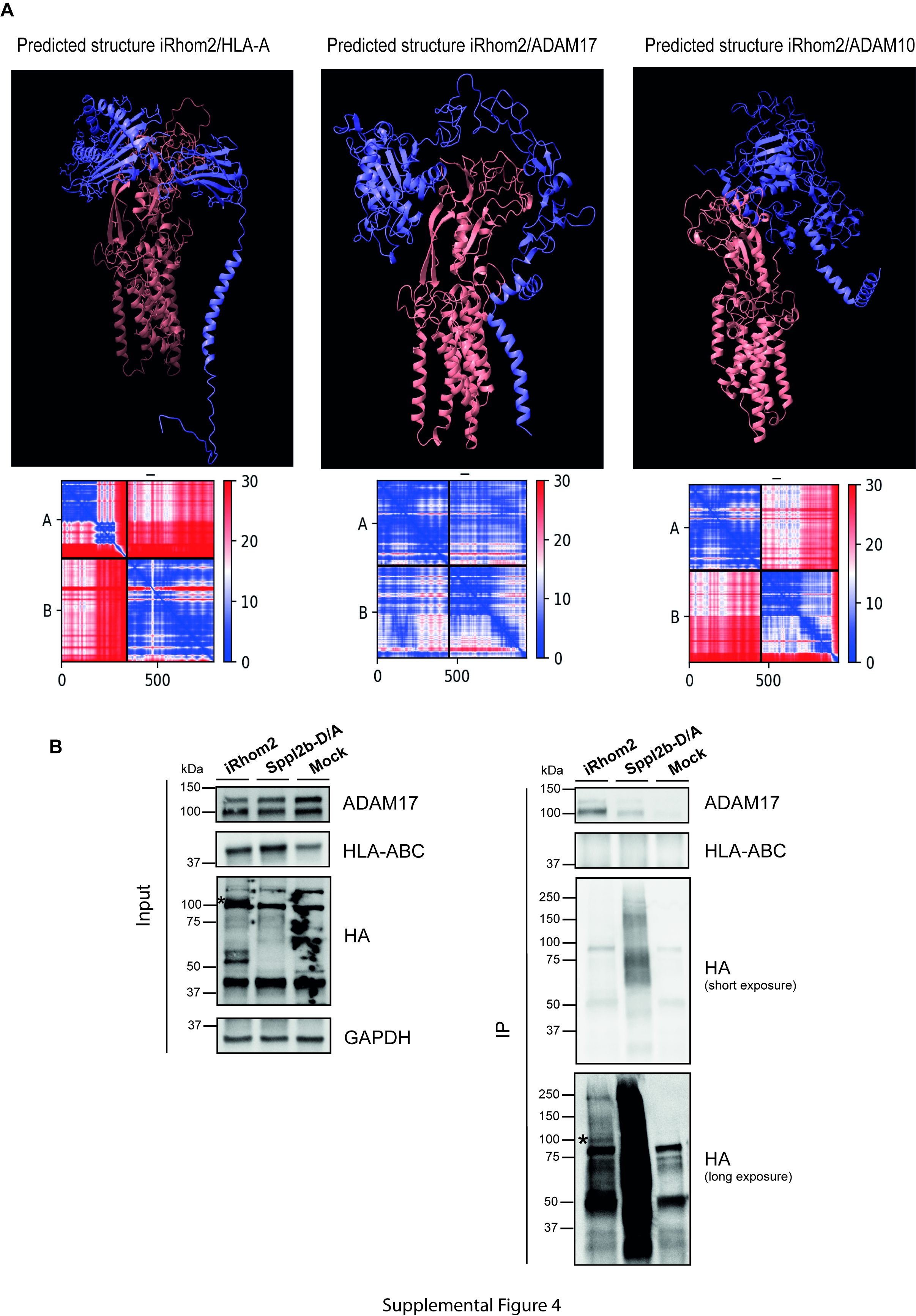

Supplement: Supplementary file 4 — Supplementary file4 Supplemental Figure 4 (A) Structural modelling of iRhom2 (in red) with HLA-A (A), ADAM17 (B) or ADAM10 (C) (in blue), using the deep learning algorithm AlphaFold 2. Conversely to the iRhom2/ADAM17 predicted interaction, the predicted align error (PAE) is low for an iRhom2/HLA-A interaction, as well as for an iRhom2/ADAM10 interaction, and therefore not supporting an interaction between iRhom2 and HLA-A. (TIF 38570 KB) [file 18_2024_5201_MOESM4_ESM.tif]
